# Research on Lateral Stability Control of Four-Wheel Independent Drive Electric Vehicle Based on State Estimation

**DOI:** 10.3390/s25020474

**Published:** 2025-01-15

**Authors:** Yu-Jie Ma, Chih-Keng Chen, Hongbin Ren

**Affiliations:** 1 Department of Vehicle Engineering, National Taipei University of Technology, Taipei 10608, Taiwan; mayujie@lyun.edu.cn; 2 School of Physics and Mechanical and Electrical Engineering, Longyan University, Longyan 364012, China; 3 School of Mechanical Engineering, Beijing Institute of Technology, Beijing 100081, China; renhongbin2106@126.com

**Keywords:** state estimation, stability criterion, control allocation

## Abstract

This paper proposes a hierarchical framework-based solution to address the challenges of vehicle state estimation and lateral stability control in four-wheel independent drive electric vehicles. First, based on a three-degrees-of-freedom four-wheel vehicle model combined with the Magic Formula Tire model (MF-T), a hierarchical estimation method is designed. The upper layer employs the Kalman Filter (KF) and Extended Kalman Filter (EKF) to estimate the vertical load of the wheels, while the lower layer utilizes EKF in conjunction with the upper-layer results to further estimate the lateral forces, longitudinal velocity, and lateral velocity, achieving accurate vehicle state estimation. On this basis, a hierarchical lateral stability control system is developed. The upper controller determines stability requirements based on driver inputs and vehicle states, switches between handling assistance mode and stability control mode, and generates yaw moment and speed control torques transmitted to the lower controller. The lower controller optimally distributes these torques to the four wheels. Through closed-loop Double Lane Change (DLC) tests under low-, medium-, and high-road-adhesion conditions, the results demonstrate that the proposed hierarchical estimation method offers high computational efficiency and superior estimation accuracy. The hierarchical control system significantly enhances vehicle handling and stability under low and medium road adhesion conditions.

## 1. Introduction

Four-wheel independent drive electric vehicles, characterized by independently controllable wheels, provide high flexibility and precise control, making them a key focus in the research and development of vehicle lateral stability control systems [1,2,3]. The lateral stability control system enhances vehicle handling performance and stability capability by continuously tracking the difference between the vehicle’s real-time operating state and a desired reference model, generating the required yaw moment, and implementing control strategies such as torque distribution adjustments across the four wheels [4,5]. Although the vehicle lateral stability control system plays a critical role in improving active safety, it is constrained by cost considerations and relies on a limited number of essential sensors to obtain data such as yaw rate, vehicle acceleration, and speed. However, key parameters that represent the vehicle’s operating state, such as the sideslip angle, and critical factors in control strategies, such as the normal forces and lateral forces on the wheels, cannot be directly measured by existing onboard sensors. These parameters are typically estimated indirectly using other measurable state information. The accuracy of this estimation is directly related to the effectiveness of the vehicle lateral stability control system [1,2,6,7,8,9,10].

A considerable amount of research has been conducted by vehicle dynamics scholars worldwide on estimating vehicle states such as the sideslip angle, wheel normal forces, and lateral forces. The estimation methods are generally divided into two main categories: state observer methods based on vehicle dynamics or kinematics models, and neural network-based methods trained on experimental data [1,6,8,10].

In the context of vehicle sideslip angle estimation, reference [9] proposes a state observer based on a discrete-time Linear Parameter-Varying (LPV) lateral vehicle dynamics model. This observer demonstrates both effective and robust estimation performance. Similarly, reference [11] utilizes an Unscented Kalman Filter (UKF) to design an observer integrating both kinematics and dynamics. A fuzzy logic controller is employed to adaptively allocate the proportional weights between the two observers, resulting in enhanced estimation accuracy. Furthermore, reference [12] proposes a method for estimating the vehicle sideslip angle using a dual neural network structure. By training the proposed neural network with a large dataset, the results indicate that this method can effectively achieve real-time estimation of the vehicle sideslip angle. In addition, reference [13] presents a sideslip angle estimation method based on a Recurrent Artificial Neural Network (RANN). Real vehicle experimental data were collected and used for training under road conditions with varying adhesion coefficients. The findings reveal that this method can adapt to changes in road adhesion coefficients while maintaining accurate sideslip angle estimation. Finally, reference [14] introduces a hybrid framework for vehicle sideslip angle estimation, combining an Unscented Kalman Filter (UKF) with a Convolutional Neural Network (CNN). The UKF leverages physical model constraints, while the CNN utilizes data-driven capabilities, achieving adaptive estimation of pseudo-measurements and their uncertainty through end-to-end training. The method dynamically adjusts process noise parameters to address model uncertainties, enhancing robustness and accuracy under nonlinear conditions.

In terms of wheel vertical loads and lateral forces, reference [15] employs a robust Proportional Multiple Integral (PMI) observer based on an LPV model. The Particle Swarm Optimization (PSO) algorithm is applied to identify the optimal solution, achieving favorable results. Reference [16] utilizes the Flat-Trac tire testing platform to collect real tire forces and accelerations along the x, y, and z axes under various testing scenarios. Through machine learning, this method can accurately estimate the tire forces in all three directions based on the input acceleration data. Reference [17] constructs a database consisting of vehicle speed, steering wheel angle, road adhesion coefficient, tire lateral forces, and longitudinal forces. Using a Long Short-Term Memory (LSTM) network for training, it achieves promising estimation results under high-adhesion road conditions during DLC tests. Reference [18] employs a three-degrees-of-freedom four-wheel vehicle model and an Adaptive Sliding Mode Observer (ASMO) to estimate the lateral forces of all four wheels. Finally, reference [19] proposes a tire force estimation method based on an Extended Kalman Filter (EKF) and a double-track vehicle model. This method considers factors such as wheel dynamics, suspension elasto-kinematics, and tire–road interactions, utilizing the Magic Formula Tire model to calculate longitudinal and lateral forces. By incorporating inputs from accelerometers, steering angle sensors, and wheel speed sensors, it achieves accurate estimation of vehicle motion states. The method emphasizes the effects of road slope and banking, as well as the adaptive updating of dynamic parameters, to enhance the accuracy and robustness of the model.

The above-mentioned large amount of research provides valuable references for this paper. References [12,13,16,17] achieve promising results in estimating vehicle sideslip angle, wheel lateral forces, and vertical loads using neural networks and machine learning methods based on databases. However, these methods heavily rely on experimental sample data, and their underlying principles are difficult to explain mathematically. Reference [11] employs a fuzzy logic controller to adaptively assign weight coefficients, fusing the two estimation values from EKF to obtain more accurate estimates. However, this method involves high computational complexity and places significant demands on the vehicle’s ECU. References [19,20,21] consider the impact of varying wheel loads under different vehicle conditions (such as passenger load, acceleration/deceleration, and turning) on the interaction forces between the wheels and the road surface, which, in turn, affect vehicle stability control. These studies estimate the vertical loads on all four wheels and subsequently estimate the wheel lateral forces and vehicle centroid sideslip angle, providing valuable insights for this work.

The primary contributions of this study are as follows:

1. A hierarchical estimation method. The upper layer uses KF and EKF observers to estimate the vertical loads on all four wheels based on data collected by low-cost onboard sensors. The lower layer focuses on a three-degrees-of-freedom four-wheel vehicle model combined with the nonlinear MF-T, utilizing an EKF observer to estimate the lateral forces on all four wheels and the vehicle centroid sideslip angle.
2. A layered architecture for vehicle lateral stability control. When the vehicle is stable, the control system provides additional yaw moments to enhance the vehicle handling performance. In contrast, when the vehicle becomes unstable, the control system generates additional yaw moments to restore stability.

The remainder of the paper is organized as follows: Section 2 details the driver model and the four-wheel vehicle dynamics model, which capture the vehicle’s dynamic characteristics, along with the linear two-degrees-of-freedom vehicle dynamics model used as a reference for controller design and the nonlinear MF-T. Section 3 presents the methodology and results of vehicle state estimation. Section 4 emphasizes the controller design, focusing on the stability criterion approach and the lower-level controller’s control allocation strategy. Section 5 discusses the experimental setup and simulation results. Finally, Section 6 provides the conclusions of the study.

## 2. Driver Model and Vehicle Dynamics Model

The driver model can simulate real driver behaviors such as steering and acceleration, serving as the foundation for studying vehicle lateral stability control [22]. In this study, the built-in driver model of CarSim 2023 was utilized for path-following tasks. An accurate vehicle dynamics model is key to reliably describing the vehicle’s dynamic performance. In our study, a four-wheel, three-degrees-of-freedom vehicle dynamics model integrated with the nonlinear MF-T was developed to represent the vehicle’s dynamic characteristics. A two-degrees-of-freedom single-track vehicle model was adopted as the reference model in the design of the vehicle controller.

### 2.1. Driver Model

**Figure 1:**
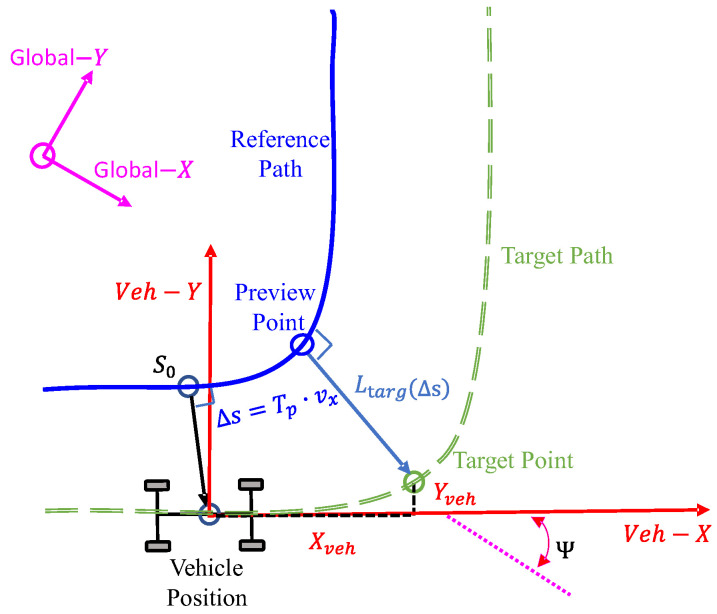
Driver model.

In this study, the CarSim built-in driver model was utilized for path following based on geometric path tracking and a single preview point to achieve stable tracking of the target path by the vehicle. As shown in Figure 1, the dark green dashed line represents the target path, the blue solid line represents the reference path, the red coordinate system corresponds to the local vehicle coordinates, and the magenta coordinate system represents the global coordinate system. The initial vehicle position was set at the origin of the local coordinate system, with the yaw angle Ψ indicating the vehicle’s orientation relative to the global coordinate system.

The reference path is defined as a continuous planar curve composed of the path distance s along the route and the corresponding lateral offset $L_{targ}\left(s\right)$. This ensures that the target path avoids sharp corners, thereby preventing abrupt vehicle turns. When the vehicle’s speed is $v_{x}$ and the preview time is $T_{p}$, the reference point $S_{0}$ is defined as the nearest point on the reference path to the vehicle’s current position. The preview point lies further along the reference path at a distance $∆s=T_{p}\cdot v_{x}$, with a lateral offset of $L_{targ}\left(∆s\right)$, and its endpoint serves as the target point. The driver model calculates the global coordinates of the target point, ($X_{targ},Y_{targ}$), using the Application Programming Interface (API) for coordinate transformations. These global coordinates are further transformed into the local vehicle coordinates ($X_{veh},Y_{veh}$) using Equations (1) and (2). Finally, the steering angle δ required for the vehicle to follow the target path is obtained using Equation (3):

(1) $$X_{veh}=X_{targ}\cdot cos(\Psi )+Y_{targ}\cdot sin(\Psi )$$

(2) $$Y_{veh}=X_{targ}\cdot sin(\Psi )+Y_{targ}\cdot cos(\Psi )$$

(3) $$\delta =\tan^{-1}\left(\frac{X_{veh}}{X_{veh}}\right)$$

### 2.2. Vehicle Dynamics Model

To accurately capture the dynamic characteristics of the vehicle, this study employed a four-wheel, three-degrees-of-freedom vehicle dynamics model integrated with the nonlinear MF-T. For the controller design, a linear two-degrees-of-freedom single-track vehicle model, which offers lower computational complexity while effectively reflecting vehicle stability and handling performance, was adopted as the reference model.

#### 2.2.1. Magic Formula Tire Model

**Figure 2:**
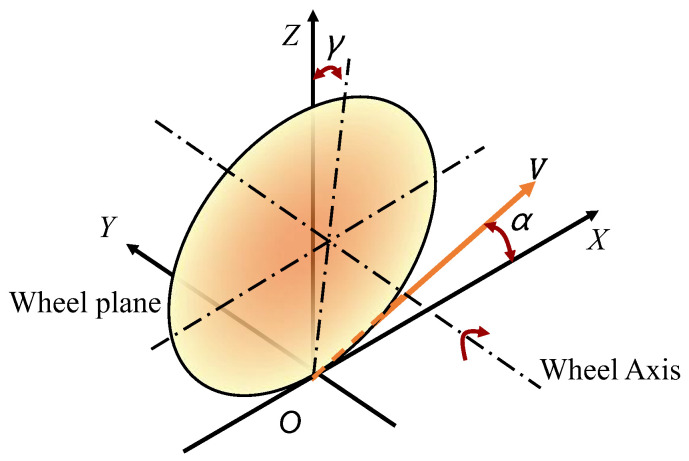
Tire slip angle α.

When a vehicle turns, due to the characteristics of the tires, the direction of wheel travel does not align with the plane of the wheel, as shown in Figure 2. The angle between them is referred to as the tire slip angle α. The lateral force $F_{y}$ exerted on the vehicle is closely related to α. When α is small and within a certain range, they are proportional. However, when α exceeds a certain threshold, the relationship becomes nonlinear [23].

**Figure 3:**
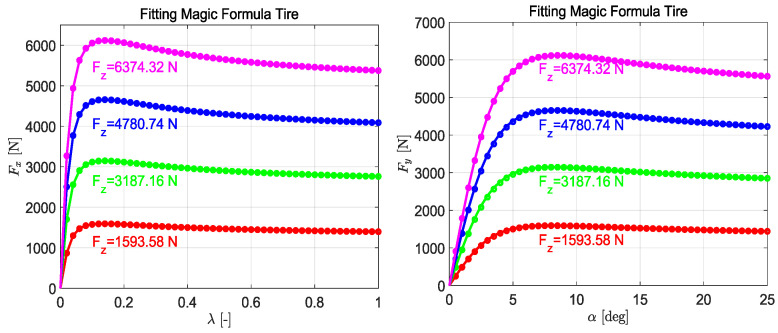
The fitted tire longitudinal force and lateral force curves.

To fully capture the nonlinear characteristics of tires, this study adopted the nonlinear MF-T, with its expression provided in (4) and (5). By fitting the model to experimental tire data from CarSim for tires of size 215/55R17, the resulting longitudinal and lateral force curves are shown in Figure 3, and the corresponding parameters in the formula are listed in Table 1. It is evident that the tire vertical load significantly affects both lateral and longitudinal forces, with both increasing as the load rises.

(4) $${F_{x}=D}_{x}\sin\left\{C_{x}\tan^{-1}\left[B_{x}\lambda -E_{x}\left(B_{x}\lambda -{tan}^{-1}\left(B_{x}\lambda \right)\right)\right]\right\}$$

(5) $$F_{y}=D_{y}\sin\left\{C_{y}\tan^{-1}\left[B_{y}\alpha -E_{y}\left(B_{y}\alpha -{tan}^{-1}\left(B_{y}\alpha \right)\right)\right]\right\}+S_{v}$$

where the parameters are defined as follows: λ represents the tire slip ratio, and α represents the tire sideslip angle. The parameters are further expressed as follows: $D_{x/y}=a_{1}{F_{z}^{2}+a_{2}F}_{z}$, $E_{x/y}=a_{6}{F_{z}^{2}+a_{7}F}_{z}+a_{8}$, $B_{x}=\frac{\left(a_{3}{F_{z}^{2}+a_{4}F}_{z}\right)e^{-a_{5}F_{z}}}{C_{x}D_{x}}$, $B_{y}=\frac{a_{3}\sin\left[a_{4}\tan^{-1}\left(a_{5}F_{z}\right)\right]}{C_{y}D_{y}}$. The effects of the camber angle are ignored, and therefore, $S_{v}=0$.

This study focuses on the vehicle’s lateral stability. Assuming a constant vehicle speed, the vehicle’s lateral force $F_{y}$ is expressed in (6).

(6) $$F_{y}=f(\mu ,F_{z},\alpha )$$

#### 2.2.2. Four-Wheel Vehicle Dynamics Model

**Figure 4:**
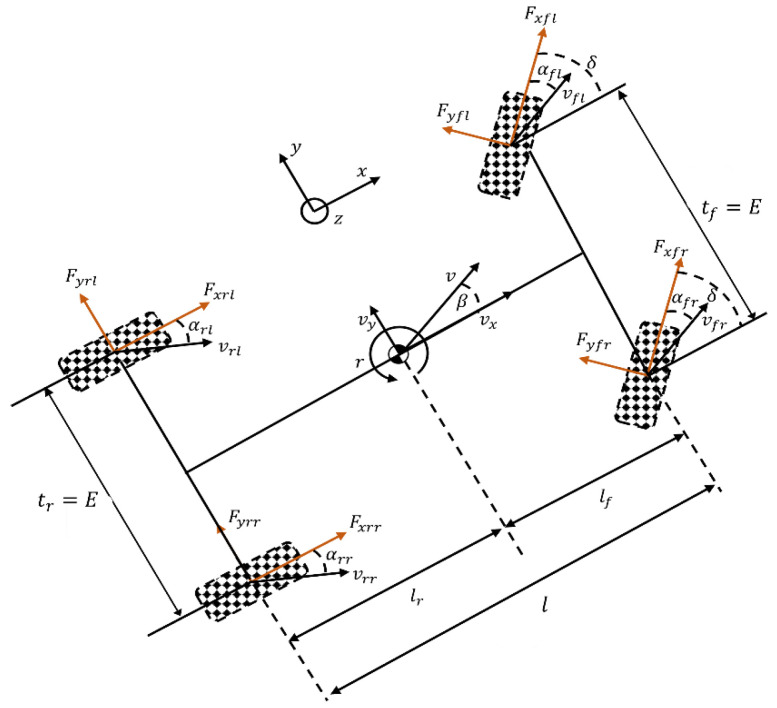
Four-wheel vehicle dynamics model.

Assuming a flat road surface and neglecting the effects of suspension and vehicle pitch motion, a four-wheel vehicle dynamics model is established as shown in Figure 4. The model includes longitudinal, lateral, and yaw motions of the vehicle. The governing equations are presented in Equations (7)–(9), where m represents the vehicle mass.

Where v represents the velocity at the vehicle’s center of gravity (CG), r is the yaw rate of the vehicle, $I_{z}$ denotes the yaw moment of inertia, and δ is the steering angle of the front wheels. $v_{x}$ and $v_{y}$ are the longitudinal and lateral velocities, respectively. $l_{f}$ and $l_{r}$ are the distances from the CG to the front and rear axles, respectively. $F_{x,ij}$ and $F_{y,ij}$ represent the longitudinal and lateral forces acting on each wheel, respectively, while $\alpha_{ij}$ denotes the slip angle of each wheel (where subscripts fl, fr, rl and rr refer to the front-left, front-right, rear-left, and rear-right wheels, respectively). Additionally, β is the sideslip angle of the vehicle body, l is the wheelbase, and $t_{f}$ and $t_{r}$ are the front and rear track widths, respectively.

(7) $$\dot{\gamma }=\frac{1}{I_{z}}\left\{\begin{array}{c} l_{f}\left[\left(F_{yfl}+F_{yfr}\right)\cos\delta +F_{xf}\sin\delta \right]-l_{r}\left(F_{yrl}+F_{yrr}\right)+\\ \frac{E}{2}\left[\left(F_{yfl}-F_{yfr}\right)\sin\delta +\left(F_{xfr}-F_{xfl}\right)\cos\delta \right] \end{array}\right\}$$

(8) $$\dot{v_{x}}=v_{y}\gamma +\frac{1}{m}\left[F_{xf}\cos\delta -\left(F_{yfl}+F_{yfr}\right)\sin\delta \right]$$

(9) $$\dot{v_{y}}={-v}_{x}\gamma +\frac{1}{m}\left[F_{xf}\sin\delta +\left(F_{yfl}+F_{yfr}\right)\cos\delta +F_{yrl}+F_{yrr}\right]$$

Since the lateral velocity of the vehicle is much smaller than the longitudinal velocity during operation, the vehicle sideslip angle β can be approximated using (10). The sideslip angles of the four wheels are expressed in (11)–(14).

(10) $$\beta =\tan^{-1}\frac{v_{y}}{v_{x}}\approx \frac{v_{y}}{v_{x}}$$

(11) $$\alpha_{fl}=\delta -\tan^{-1}\left(\frac{v_{y}+l_{f}\gamma }{v_{x}-0.5\cdot E\gamma }\right)$$

(12) $$\alpha_{fr}=\delta -\tan^{-1}\left(\frac{v_{y}+l_{f}\gamma }{v_{x}+0.5\cdot E\gamma }\right)$$

(13) $$\alpha_{rl}=-\tan^{-1}\left(\frac{v_{y}-l_{f}\gamma }{v_{x}-0.5\cdot E\gamma }\right)$$

(14) $$\alpha_{rr}=-\tan^{-1}\left(\frac{v_{y}-l_{f}\gamma }{v_{x}+0.5\cdot E\gamma }\right)$$

#### 2.2.3. Linear Two-Degrees-of-Freedom Vehicle Dynamics Model

**Figure 5:**
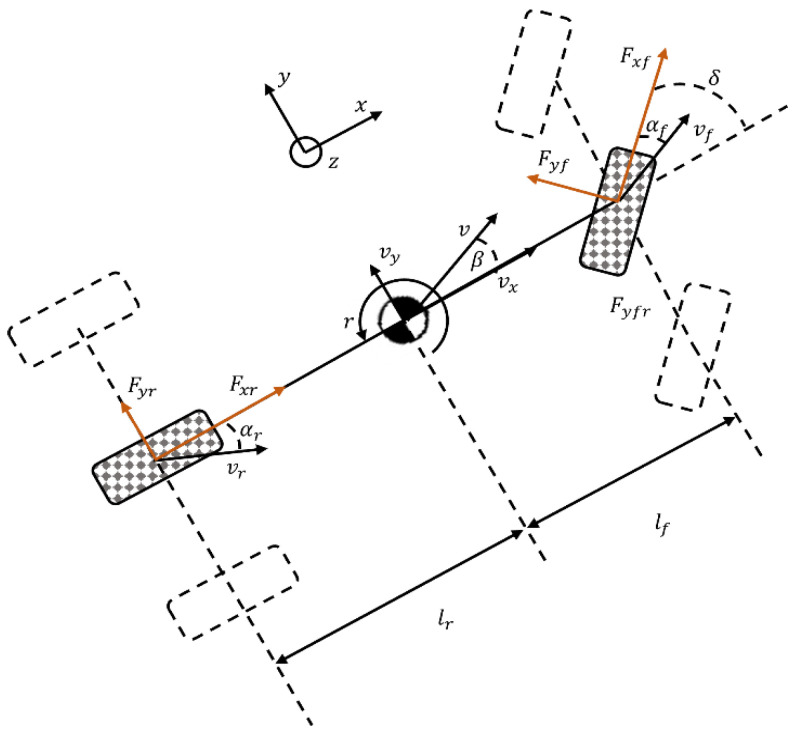
Linear two-degrees-of-freedom vehicle dynamics model.

Assuming the front-wheel steering angle δ is small, the relationship between tire slip angle and cornering stiffness can be approximated as linear, as shown in (15) and (16) [23]. By combining the left and right wheels on both the front and rear axles in Figure 4 and using small-angle approximations, a simplified reference model representing vehicle stability capability and handling performance was derived. This reference model, required for the controller, is illustrated in Figure 5. Here, $C_{\alpha f}$ and $C_{\alpha r}$ represent the cornering stiffness of the front and rear wheels, respectively, and $\alpha_{f}$ and $\alpha_{r}$ denote the slip angles of the front and rear wheels, respectively, as shown in (17) and (18). Through dynamic analysis, the state-space equations for this reference model were obtained, as expressed in (19).

(15) $$F_{yf}=C_{\alpha f}\times \alpha_{f}$$

(16) $$F_{yr}=C_{\alpha r}\times \alpha_{r}$$

(17) $$\alpha_{f}=\delta -\beta -\frac{l_{f}r}{v_{x}}$$

(18) $$\alpha_{r}=\beta -\frac{l_{r}r}{v_{x}}$$

(19) $$\dot{x}=Ax+Bu+Gw$$

where $x=\left[\begin{array}{c} \beta \\ r \end{array}\right]$, $u=M_{z}$, w=δ, $A=\left[\begin{array}{cc} a_{11} & a_{12}\\ a_{21} & a_{22} \end{array}\right]=\left[\begin{array}{cc} -\frac{\left(C_{\alpha f}+C_{\alpha r}\right)}{mv_{x}} & \frac{l_{r}C_{\alpha r}-l_{f}C_{\alpha f}}{m{v_{x}}^{2}}-1\\ \frac{l_{r}C_{\alpha r}-l_{f}C_{\alpha f}}{I_{z}} & -\frac{\left({l_{f}^{2}C}_{\alpha f}+{l_{r}^{2}C}_{\alpha r}\right)}{I_{z}v_{x}} \end{array}\right]$,$B=\left[\begin{array}{c} b_{1}\\ b_{2} \end{array}\right]=\left[\begin{array}{c} 0\\ \frac{1}{I_{z}} \end{array}\right]$, $G=\left[\begin{array}{c} g_{1}\\ g_{2} \end{array}\right]=\left[\begin{array}{c} \frac{C_{\alpha f}}{mv_{x}}\\ \frac{l_{f}C_{\alpha f}}{I_{z}} \end{array}\right]$.

## 3. Vehicle State Estimation

To enable the estimation of vehicle states, it is assumed that the road adhesion coefficient μ is known. The onboard Inertial Measurement Unit (IMU) measures the vehicle’s longitudinal acceleration $a_{x}$, lateral acceleration $a_{y}$, yaw rate γ, and roll rate $\dot{\theta }$. Suspension deflection sensors measure the suspension deflection $\Delta s_{ij}$, and wheel speed sensors measure the angular velocities of the four wheels $\omega_{ij}$. Based on the method proposed in [21], a hierarchical estimator is developed. In the upper layer, a state equation and a measurement equation are established based on data measured by existing low-cost onboard sensors. The Kalman Filter (KF) and Extended Kalman Filter (EKF) observers are employed to estimate the wheel normal force $F_{zij}$. In the lower layer, a three-degrees-of-freedom, four-wheel vehicle model and the nonlinear MF-T model are used as the research objects. Combined with signals measured by wheel speed sensors, the IMU, and other sensors, the system’s state equation and measurement equation are constructed. The EKF observer is then utilized to estimate the wheel lateral force $F_{yij}$, longitudinal velocity $v_{x}$, lateral velocity $v_{y}$, and ultimately the vehicle’s centroid sideslip angle β.

### 3.1. Vehicle Vertical Load $F_{zij}$ Estimation

During vehicle operation, the loads on the four wheels vary due to driving conditions such as acceleration or deceleration (load transfer between the front and rear axles) and cornering (load transfer from the inner to the outer wheels). These load variations significantly affect the lateral forces on the tires, which in turn influence the vehicle’s lateral stability. Typically, by neglecting the effects of suspension and vehicle pitch, the vertical loads on each wheel are calculated based on the vehicle’s static load distribution and considering load transfer, as shown in (20)–(23), where $h_{c}$ represents the height of the vehicle’s CG. This method is referred to as open-loop estimation. However, since open-loop estimation assumes a fixed CG position during load transfer and neglects the coupling between longitudinal and lateral motions, it cannot accurately and dynamically represent the vertical loads on each wheel during vehicle operation.

(20) $$F_{zfl}=\frac{ml_{r}}{2l}g-\frac{mh_{c}}{2l}a_{x}-\frac{ml_{r}h_{c}}{lt_{f}}a_{y}$$

(21) $$F_{zr}=\frac{ml_{r}}{2l}g-\frac{mh_{c}}{2l}a_{x}+\frac{ml_{r}h_{c}}{lt_{f}}a_{y}$$

(22) $$F_{zrl}=\frac{ml_{f}}{2l}g+\frac{mh_{c}}{2l}a_{x}-\frac{ml_{f}h_{c}}{lt_{r}}a_{y}$$

(23) $$F_{zrr}=\frac{ml_{f}}{2l}g+\frac{mh_{c}}{2l}a_{x}+\frac{ml_{f}h_{c}}{lt_{r}}a_{y}$$

To improve the estimation of vertical loads on the wheels, this study utilizes KF and EKF to estimate the vertical loads $F_{zij}$ on the wheels. First, based on the lateral acceleration $a_{y}$ measured by the onboard IMU sensors and the suspension deflection $\Delta s_{ij}$ measured by the suspension deflection sensors, the transient lateral load transfer ${\Delta F}_{zlt}$ and the vehicle roll angle θ are calculated using (24)–(26). Next, linear state equations and measurement equations, as shown in (27), are established to estimate the lateral load transfer ${\Delta F}_{zl}$ using the KF. Finally, considering the coupling relationship between the vehicle’s longitudinal and lateral motions, the nonlinear state equations and measurement equations, as shown in (30), are developed. Using the EKF, the vertical loads $F_{zij}$ on all four wheels are calculated.

(24) $$I_{xx}\ddot{\theta }+C_{R}\dot{\theta }+K_{R}\theta =ma_{y}h_{c}+mh_{c}gsin\left(\theta \right)$$

(25) $${\Delta F}_{zl}=-2\left(\frac{k_{f}}{t_{f}}+\frac{k_{r}}{t_{r}}\right)\theta -2m_{s}\frac{a_{y}}{l}\left(\frac{l_{r}h_{f}}{t_{f}}+\frac{l_{f}h_{r}}{t_{r}}\right)$$

(26) $$\theta =\frac{\left({\Delta s}_{fl}-{\Delta s}_{fr}+{\Delta s}_{rl}-{\Delta s}_{rr}\right)}{2E}$$

(27) $$\begin{array}{l} X_{1,k}=A_{1}X_{1,k-1}+w_{1,k}\\ Y_{1,k}=H_{1}X_{1,k}+v_{1,k} \end{array}$$

where *k* and *k* − 1 represent the sampling time steps. The state variable $X_{1}$ is defined as the follows: $X_{1}={\left(\begin{array}{cccc} x_{11} & x_{12} & \cdots & x_{15} \end{array}\right)}^{T}={\left[∆F_{zl},a_{y},{\dot{a}}_{y},\theta ,\dot{\theta }\right]}^{T}$, the state transition matrix is represented as (28), $t_{e}$ represents the sampling time, and $K_{R}$ and $C_{R}$ denote the total spring stiffness and total damping coefficient during vehicle roll, respectively. $I_{xx}$ is the roll moment of inertia of the sprung mass about the x-axis, while $k_{f}$ and $k_{r}$ represent the roll stiffness of the front and rear axles, respectively. The measurement variable is defined as $Y_{1}={\left(\begin{array}{cccc} y_{11} & y_{12} & y_{13} & y_{14} \end{array}\right)}^{T}={\left[a_{y},\theta ,\dot{\theta },∆F_{zl}\right]}^{T},$ and the observation matrix $H_{1}$ is expressed as (29). $w_{1,k}$ and $v_{1,k}$ denote the process and measurement noise, respectively. Both have a mean value of zero, with covariance matrices labeled as $Q_{1}$ and $R_{1}$.

(28) $$A_{1}=\left(\begin{array}{ccccc} 1 & 0 & \frac{-{2t}_{e}m\left(l_{r}h_{f}+l_{f}h_{r}\right)}{lE} & 0 & \frac{-{2t}_{e}\left(k_{f}+k_{r}\right)}{E}\\ 0 & 1 & t_{e} & 0 & 0\\ 0 & 0 & 1 & 0 & 0\\ 0 & 0 & 0 & 1 & t_{e}\\ 0 & \frac{t_{e}mh_{c}}{I_{xx}} & 0 & \frac{t_{e}\left(m{gh}_{c}-K_{R}\right)}{I_{xx}} & 1+\frac{t_{e}\left(-C_{R}\right)}{I_{xx}} \end{array}\right)$$

(29) $$H_{1}=\left[\begin{array}{cccc} 0 & 1 & g & 0\\ 0 & 0 & 1 & 0\\ 0 & 0 & 0 & 1\\ 1 & 0 & 0 & 0 \end{array}\right]$$

(30) $$\begin{array}{l} X_{2,k}=f_{2,k-1}(X_{2,k-1})+w_{2,k}\\ Y_{2,k}=h_{2}{(X}_{2,k})+v_{2,k} \end{array}$$

Here, the state variable $X_{2}={\left(\begin{array}{cccc} x_{21} & x_{22} & \cdots & x_{28} \end{array}\right)}^{T}={\left[F_{zfl},F_{zfr},F_{zrl},F_{zrr},a_{x},{\dot{a}}_{x}{,a}_{y},{\dot{a}}_{y}\right]}^{T}$. The nonlinear state transition function $f_{2}(X_{2})$, describing the relationship between the state at sampling time k and the previous time k−1, is expressed in (31). The corresponding state transition matrix $A_{2,k-1}$ is derived in (32) and $n_{1}=\frac{mh_{c}t_{e}}{2l}$, $n_{2}=\frac{m{l_{r}h}_{c}t_{e}}{El}$, $n_{3}=\frac{mh_{c}^{2}t_{e}}{Elg}$, $n_{4}=\frac{mh_{c}l_{f}t_{e}}{El}$. The measured values $Y_{2}={\left(\begin{array}{cccc} y_{21} & y_{22} & \cdots & y_{25} \end{array}\right)}^{T}={\left[{∆F_{zl},(F}_{zfl}{+F}_{zfr}),a_{x},a_{y},mg\right]}^{T}$. The measurement function $h_{2}\left(X_{2}\right)$ is expressed in (33), and its corresponding observation matrix $H_{2}$ is derived in (34). The process noise $w_{2,k}$ and measurement noise $v_{2,k}$ are both zero-mean and have covariances $Q_{2}$ and $R_{2}$, respectively.

(31) $$\begin{array}{l} \begin{array}{l} x_{21,k}=x_{21,k-1}-n_{1}x_{26,k-1}-n_{2}x_{28,k-1}+n_{3}\left(x_{25,k-1}x_{28,k-1}+x_{26,k-1}x_{27,k-1}\right)\\ x_{22,k}=x_{22,k-1}-n_{1}x_{26,k-1}+n_{2}x_{28,k-1}-n_{3}\left(x_{25,k-1}x_{28,k-1}+x_{26,k-1}x_{27,k-1}\right)\\ x_{23,k}=x_{23,k-1}+n_{1}x_{26,k-1}-n_{4}x_{28,k-1}-n_{3}\left(x_{25,k-1}x_{28,k-1}+x_{26,k-1}x_{27,k-1}\right) \end{array}\\ \begin{array}{l} x_{24,k}=x_{24,k-1}+n_{1}x_{26,k-1}+n_{4}x_{28,k-1}+n_{3}\left(x_{25,k-1}x_{28,k-1}+x_{26,k-1}x_{27,k-1}\right)\\ x_{25,k}=x_{25,k-1}+t_{e}x_{26,k-1}\\ x_{26,k}=x_{26,k-1}\\ x_{27,k}=x_{27,k-1}+t_{e}x_{28,k-1}\\ x_{28,k}=x_{28,k-1} \end{array} \end{array}$$

(32) $$A_{2,k-1}=\frac{\partial f_{2}\left(X_{2,k-1}\right)}{\partial X_{2}}$$

(33) $$\begin{array}{l} y_{21,k}=x_{21,k}-x_{22,k}+x_{23,k}-x_{24,k}\\ y_{22,k}=x_{21,k}+x_{22,k}\\ y_{23,k}=x_{25,k}\\ y_{24,k}=x_{27,k}\\ y_{25,k}=x_{21,k}+x_{22,k}+x_{23,k}+x_{24,k} \end{array}$$

(34) $$H_{2}=\frac{\partial h_{2}\left(X_{2,k}\right)}{\partial X_{2}}$$

### 3.2. Vehicle Lateral Force and Sideslip Angle Estimation

The lateral forces $F_{yij}$ on the four wheels can be calculated from (35)–(40), which is referred to as open-loop estimation [21]. However, during vehicle operation, the position of the CG changes with driving conditions. Additionally, $a_{y}$ and $\dot{\gamma }$ measured by the IMU are inevitably affected by external noise, leading to inaccuracies in the estimated lateral forces. To address this issue, this paper employs an EKF to estimate $F_{yij}$, as well as $v_{x}$ and $v_{y}$ of the vehicle, thereby estimating the sideslip angle β.

(35) $$F_{yf}=\frac{ma_{y}l_{r}-I_{z}\dot{\gamma }}{l\cos\delta }$$

(36) $$F_{yr}=\frac{ma_{y}l_{f}+I_{z}\dot{\gamma }}{l}$$

(37) $$F_{yfl}=\frac{F_{z,fl}}{F_{z,fl}+F_{z,fr}}F_{yf}$$

(38) $$F_{yfr}=\frac{F_{z,fr}}{F_{z,fl}+F_{z,fr}}F_{yf}$$

(39) $$F_{yrl}=\frac{F_{z,rl}}{F_{z,rl}+F_{z,rr}}F_{yr}$$

(40) $$F_{yrr}=\frac{F_{z,rr}}{F_{z,rl}+F_{z,rr}}F_{yr}$$

The angular velocities $\omega_{ij}$ of the four wheels are measured by the wheel speed sensors, and the vehicle’s longitudinal velocity ${\hat{v}}_{x}$ is calculated using (41) and R represents the wheel radius. Combined with the vertical loads $F_{zij}$ estimated by the upper layer, the nonlinear state and measurement equations are derived based on the four-wheel vehicle dynamics model and the MF-T, as described in (6)–(9). These equations are expressed in (42). The EKF is then employed to estimate the lateral forces $F_{yij}$, longitudinal velocity $v_{x}$, and lateral velocity $v_{y}$. Finally, the vehicle’s sideslip angle β is determined through (43).

(41) $${\hat{v}}_{x}=\frac{\left(\omega_{fl}+\omega_{fr}+\omega_{rl}+\omega_{rr}\right)}{4}R$$

(42) $$\begin{array}{l} X_{3,k}=f_{3,k-1}(X_{3,k-1},U_{3,k-1})+w_{3,k}\\ Y_{3,k}=h_{3}{(X}_{3,k})+v_{3,k} \end{array}$$

(43) $$\beta =\tan^{-1}\left(\frac{v_{y}}{v_{x}}\right)$$

Here, the state variables $X_{3}={\left(\begin{array}{cccc} x_{31} & x_{32} & \cdots & x_{38} \end{array}\right)}^{T}={\left[\gamma ,v_{x},v_{y},F_{yfl},F_{yfr},F_{yrl},F_{yrr},F_{xf}\right]}^{T}$, with the control input variables $U_{3}={\left(\begin{array}{cccc} u_{31} & u_{32} & \cdots & u_{35} \end{array}\right)}^{T}={\left[\delta ,F_{zfl},F_{zfr},F_{zrl},F_{zrr}\right]}^{T}$. The nonlinear state transition function $f_{3}(X_{3})$, which describes the relationship between the state at sampling time k and the previous time k−1, is expressed in (44). Since (44) includes $F_{yij,k-1}$, the corresponding state transition matrix $A_{3,k-1}$ is derived using (6), (11) and the vertical loads $F_{zij}$ estimated in the upper layer. This derivation is carried out using MATLAB’s symbolic computation tools, such as syms and jacobian. The measurement vector is $Y_{3}={\left(\begin{array}{cccc} y_{31} & y_{32} & y_{33} & y_{34} \end{array}\right)}^{T}={\left[\gamma ,{\hat{v}}_{x},a_{x},a_{y}\right]}^{T}$, with the measurement function $h_{3}\left(X_{3}\right)$ provided in (45). Its corresponding observation matrix $H_{3}$ is derived in (46). The process noise $w_{3,k}$ and measurement noise $v_{3,k}$ both have a mean value of zero, and their covariance matrices are denoted as $Q_{3}$ and $R_{3}$, respectively.

(44) $$\begin{array}{l} x_{31,k}=x_{31,k-1}+\frac{t_{e}}{I_{z}}\left\{\begin{array}{c} \begin{array}{c} l_{f}\left[\cos\left(u_{31,k}\right)\left(x_{34,k-1}++x_{35,k-1}\right)+\sin\left(u_{31,k}\right)x_{38,k-1}\right]-\\ l_{r}\left(x_{36,k-1}+x_{37,k-1}\right)+\frac{E}{2}\sin\left(u_{31,k}\right)\left(x_{34,k-1}-x_{35,k-1}\right)+ \end{array}\\ \frac{E}{2}\left[\cos\left(u_{31,k}\right)x_{38,k-1}\left(u_{33,k-1}-u_{31,k-1}\right)/\left(u_{33,k-1}+u_{31,k-1}\right)\right] \end{array}\right\}\\ x_{32,k}=x_{32,k-1}+t_{e}x_{31,k-1}x_{33,k-1}+\frac{t_{e}}{m}\left[\begin{array}{c} x_{38,k-1}\cos\left(u_{31,k}\right)\\ -\left(x_{34,k-1}+x_{35,k-1}\right)\sin\left(u_{31,k}\right) \end{array}\right]\\ x_{33,k}=x_{33,k-1}+\frac{t_{e}}{m}\left[\begin{array}{c} x_{38,k-1}\sin\left(u_{31,k}\right)+\\ \left(x_{34,k-1}+x_{35,k-1}\right)\cos\left(u_{31,k}\right)+\\ x_{36,k-1}+x_{37,k-1} \end{array}\right]-t_{e}x_{31,k-1}x_{32,k-1}\\ x_{34,k}=x_{34,k-1}+{t_{e}x}_{32,k-1}\left(-x_{34,k-1}+F_{yfl,k-1}\right)\\ x_{35,k}=x_{35,k-1}+{t_{e}x}_{32,k-1}\left(-x_{35,k-1}+F_{yfr,k-1}\right)\\ x_{36,k}=x_{36,k-1}+{t_{e}x}_{32,k-1}\left(-x_{36,k-1}+F_{yrl,k-1}\right)\\ x_{37,k}=x_{37,k-1}+{t_{e}x}_{32,k-1}\left(-x_{37,k-1}+F_{yrr,k-1}\right)\\ x_{38,k}=x_{38,k-1} \end{array}$$

(45) $$\begin{array}{l} y_{31,k}=x_{31,k}\\ y_{32,k}=x_{32,k}\\ y_{33,k}=\frac{\left[-\left(x_{34,k}+x_{35,k}\right)\sin\left(u_{31,k}\right)+x_{38,k}\cos\left(u_{31,k}\right)\right]}{m}\\ y_{34,k}=\frac{\left[\left(x_{34,k}+x_{35,k}\right)\cos\left(u_{31,k}\right)+\left(x_{36,k}+x_{37,k}\right)+x_{36,k}\sin\left(u_{31,k}\right)\right]}{m} \end{array}$$

(46) $$H_{3,k}=\frac{\partial h_{3}\left(X_{3,k}\right)}{\partial X_{3}}$$

### 3.3. Cornering Stiffness Estimation

As noted in Section 2.2.3, when α is small, the slip angle and cornering stiffness exhibit a linear relationship. However, during vehicle operation, due to varying driving conditions, the vertical load on the wheels changes dynamically, leading to fluctuations in the vehicle’s cornering stiffness. To achieve the real-time estimation of cornering stiffness and lay the foundation for subsequent research on vehicle stability, this study utilizes the $F_{yij}$ and β obtained in Section 3.2. A KF is employed for the estimation process. Based on (15)–(18), the corresponding state-space and observation equations are derived as follows:

(47) $$\begin{array}{l} X_{k}=AX_{k-1}+w_{k}\\ Y_{k}=CX_{k-1}+v_{k} \end{array}$$

Here, the state variable is $X={\left(C_{\alpha f}C_{\alpha r}\right)}^{T}$, and the measurement variable is $Y={\left(F_{yf}F_{yr}\right)}^{T}={\left(\begin{array}{cc} \left(F_{yfl}+F_{yfr}\right) & \left(F_{yrl}+F_{yrr}\right) \end{array}\right)}^{T}$, The state transition matrix $A=I_{2}$ (identity matrix of size 2), and the observation matrix C, as shown in (48), is derived from (16) and (17). The process noise $w_{k}$ and measurement noise $v_{k}$ are both zero-mean, and their covariance matrices are denoted as Q and R, respectively.

(48) $$C=\left[\begin{array}{cc} \alpha_{f} & 0\\ 0 & \alpha_{r} \end{array}\right]$$

### 3.4. Estimation Results

**Figure 6:**
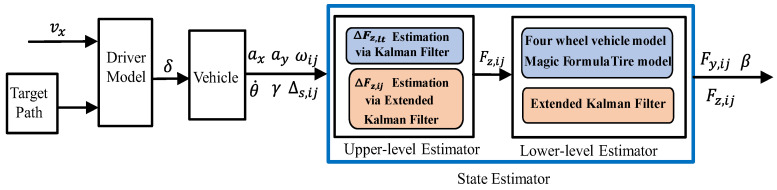
Block diagram of the estimation.

To verify the accuracy and reliability of the proposed method for estimating $F_{zij}$ and $F_{yij}$, and β, as well as the reliability of cornering stiffness estimation, we conducted DLC tests under three different road adhesion conditions (low, medium, and high). The tests were performed using co-simulation between CarSim, MATLAB (2023b), and Simulink, with a C-class hatchback vehicle equipped with four in-wheel motors, each providing power to an individual wheel. The driver’s preview time was 0.58 s, and the block diagram of the overall estimator structure is shown in Figure 6. The main parameters of the vehicle are listed in Table 2. In the tests, the open-loop estimation method is denoted as O-Est, the proposed method using EK and EKF is denoted as Est, and the direct outputs from CarSim, serving as the ground truth, are denoted as Carsim.

#### 3.4.1. Estimation Results of $F_{zij}$

**Figure 7:**
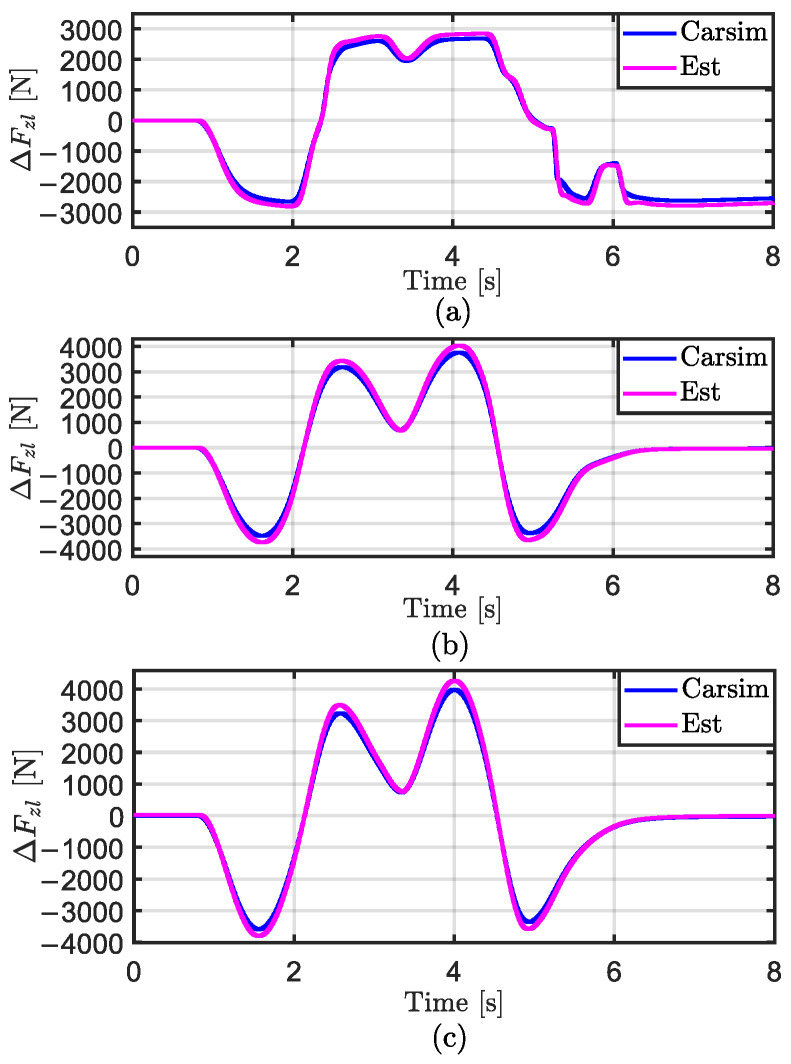
Estimation results of $∆F_{zl}$ in the DLC test at $v_{x}$ = 80 km/h. (**a**) μ=0.3; (**b**) μ=0.5; (**c**) μ=0.85.

In Figure 7, the lateral load transfer $∆F_{zl}$ estimated by the upper layer of the proposed hierarchical estimator, is labeled as “Est”, while the results directly calculated by CarSim are labeled as “Carsim”. From the figure, it can be observed that the $∆F_{zl}$ estimated using the “Est” method closely aligns with the true values output by CarSim under most operating conditions, demonstrating its reliability as an input for the lower-layer estimator. Referring to Table 3, under low road adhesion coefficients, due to vehicle instability in the later stages, the estimated results show a significant deviation from the true values, with a maximum absolute error (ME) of 534.431 N and a root mean square error (RMSE) of 145.243 N. Under medium and high road adhesion coefficients, the $∆F_{zl}$ estimated using the “Est” method closely follows the true values. However, when the vehicle’s lateral acceleration $a_{y}>0.3\;g$ due to sharp steering maneuvers, the error increases. The ME reaches 297.750 N and 285.897 N, respectively, while the RMSE is 150.768 N and 126.757 N, respectively.

**Figure 8:**
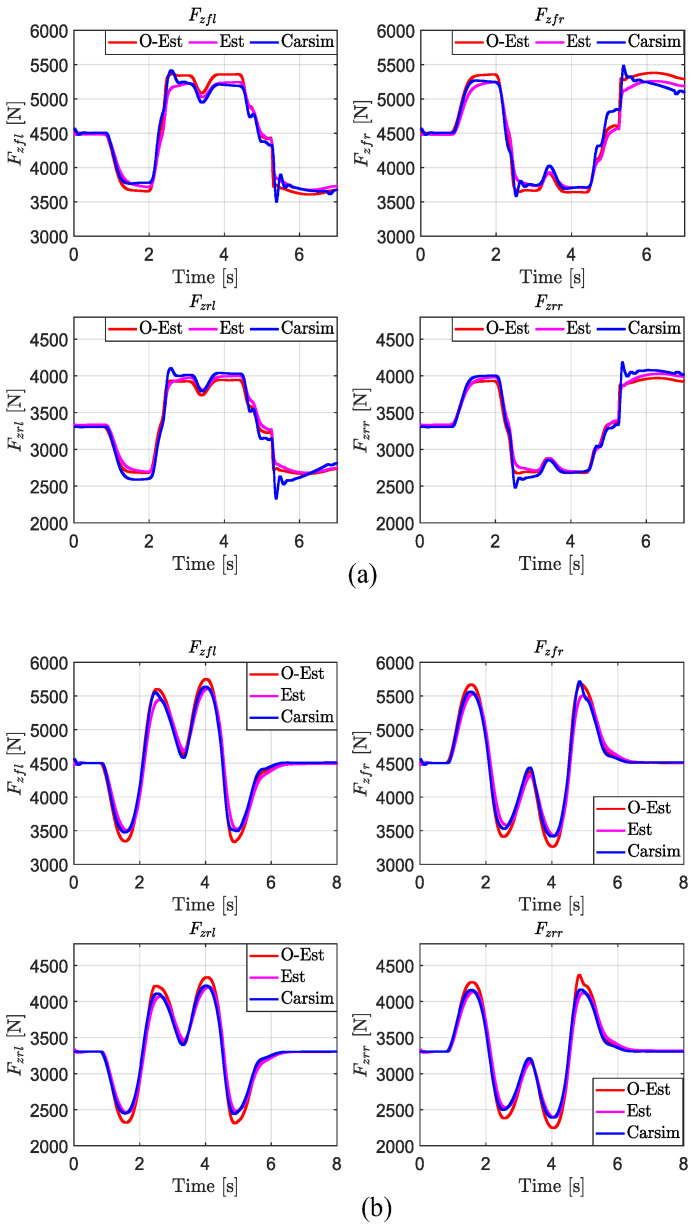

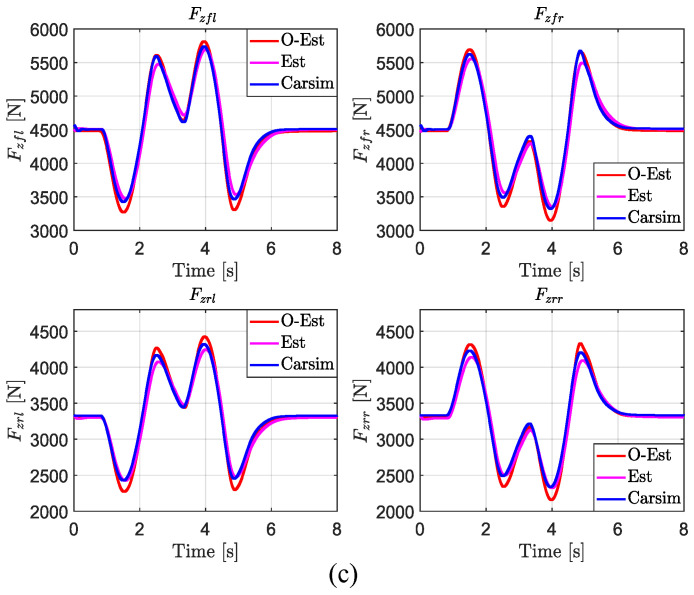
Estimation results of $F_{zij}$ in the DLC test at $v_{x}$ = 80 km/h. (**a**) μ=0.3; (**b**) μ=0.5; (**c**) μ=0.85.

**Figure 9:**
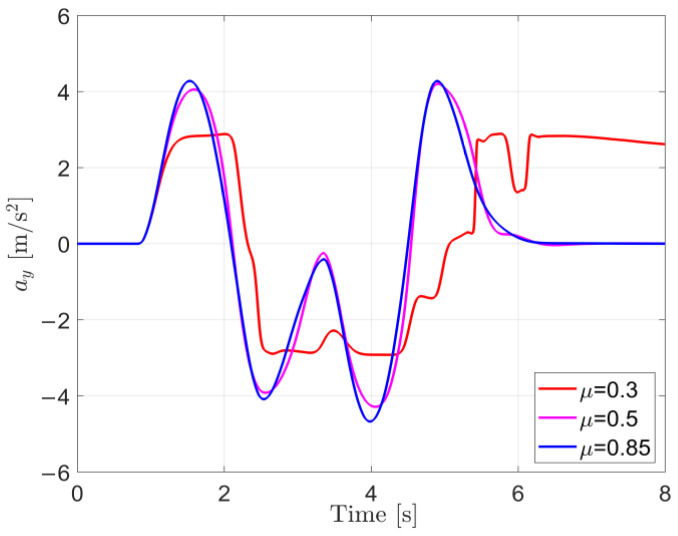
Lateral acceleration $a_{y}$ under diffident μ in the DLC test at $v_{x}$ = 80 km/h.

From Figure 8, it can be observed that under low-, medium-, and high-road-adhesion conditions, both the O-Est and Est methods estimate $F_{zij}$ well and closely follow the true values output by CarSim in most scenarios. However, under certain extreme conditions, the O-Est method shows significant deviation from the true values. Combined with the analysis of Figure 9, it can be seen that under low-adhesion conditions (μ=0.3), when the vehicle’s lateral acceleration $a_{y}$ approaches μg (indicating the onset of vehicle sliding), the deviation of the O-Est method increases significantly, while the Est method continues to closely track the true values. On medium- and high-adhesion roads (μ=0.5 and μ=0.85), when the vehicle’s lateral acceleration $a_{y}>0.3\;g$, the O-Est method begins to deviate from the true values output by CarSim, whereas the Est method consistently follows the true values accurately. Analysis indicates that this is primarily due to the change in the vehicle’s center of mass position during intense motion, as well as the O-Est method’s inherent neglect of the coupling effect between lateral and longitudinal accelerations. Consequently, the O-Est method shows deviations under intense vehicle motion, whereas the Est method, leveraging EKF estimation, effectively tracks the true values across all conditions.

From the error data for $F_{zij}$ estimation presented in Table 4, it is evident that the Est method provides more accurate estimations of $F_{zij}$ compared to the O-Est method. This is particularly notable under low-adhesion conditions (μ=0.3), where the Est method improves the estimation results in terms of mean absolute error (MAE), ME, and RMSE by 59.34%, 44.59%, and 52.52%, respectively, compared to the O-Est method. Using the Profile tool in MATLAB (2023b) to analyze execution times, both methods complete their computations within 0.1 s. During runtime, the estimator is called 1606 times, with each estimation taking $6\times 10^{-5}$ s, which is significantly shorter than the typical control cycle time of $5\times 10^{-3}$ s for mainstream controllers. Therefore, the Est method demonstrates higher computational accuracy and reliable performance.

#### 3.4.2. Estimation Results of $F_{yij}$ and β

**Figure 10:**
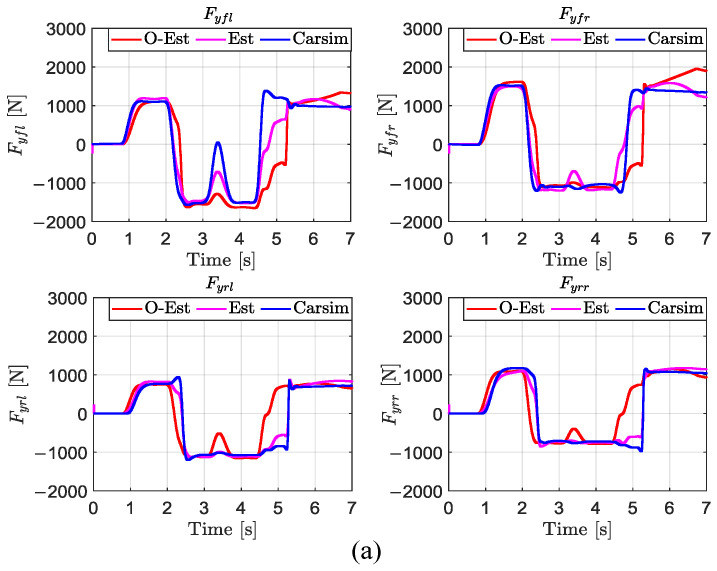

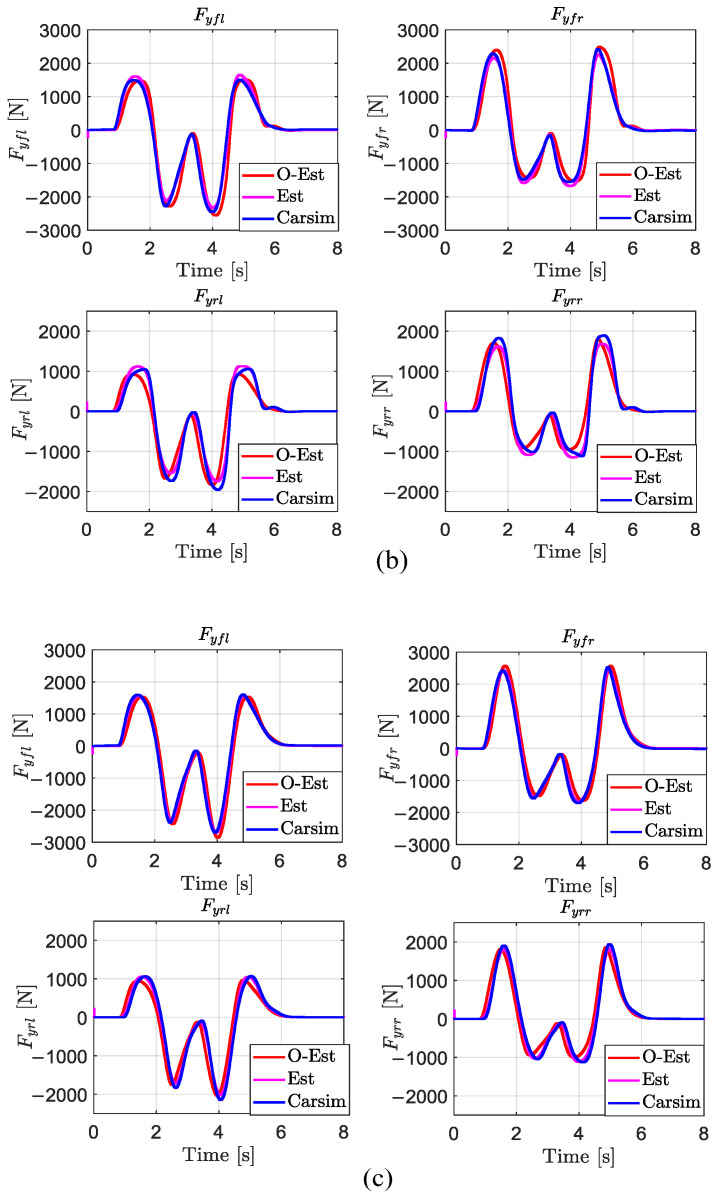
Estimation of $F_{yij}$ in the DLC test with $v_{x}$ = 80 km/h. (**a**) μ=0.3; (**b**) μ=0.5; (**c**) μ=0.85.

From the estimation results shown in Figure 10, under low-road-adhesion conditions, both estimation methods exhibit deviations from the true values in the later stages of the test, with the O-Est method showing overall larger deviations compared to the Est method. Under medium- and high-road-adhesion conditions, both the O-Est and Est methods can generally estimate $F_{yij}$ accurately, closely following the true values in most scenarios. However, significant deviations in the O-Est method occur at 0.8 s and 4 s. By analyzing the lateral acceleration shown in Figure 9, it can be observed that under low-adhesion conditions (μ=0.3), when the vehicle’s lateral acceleration $a_{y}>\mu g$, indicating the onset of sliding and intense motion, the vehicle’s center of mass position changes significantly. This leads to larger deviations in the $F_{yij}$ estimated by the O-Est method. In contrast, although the Est method relies on the vertical load $F_{zij}$ values estimated by the upper layer, which may have larger errors, the use of EKF improves the accuracy of $F_{yij}$ estimation to some extent. Under medium- and high-road-adhesion conditions, during periods of intense vehicle motion, significant changes in the center of mass position also occur. This results in larger deviations in the $F_{yij}$ estimated by the O-Est method at 0.8 s and 4 s.

From the data in Table 5, it is clear that the Est method provides more accurate $F_{yij}$ estimations compared to the O-Est method. Under low-adhesion conditions (μ=0.3), the Est method significantly outperformed the O-Est method in terms of estimation accuracy. The MAEs for the two methods are 70.56 N and 143.03 N, respectively; the MEs are 512.96 N and 1282.69 N, respectively; and the RMSEs are 109.94 N and 274.26 N, respectively. The Est method improved the estimation accuracy compared to O-Est by 50.67%, 60.01%, and 59.91% in terms of the MAE, ME and RMSE, respectively. Using the Profile tool to analyze execution times, both methods completed their computations within 0.19 s. During the runtime, the estimator was called 1606 times, with each estimation taking $1.18\times 10^{-4}$ s, which is shorter than the typical control cycle time of $5\times 10^{-3}$ s for mainstream controllers. Therefore, the Est method demonstrates higher computational accuracy and reliable performance.

**Figure 11:**
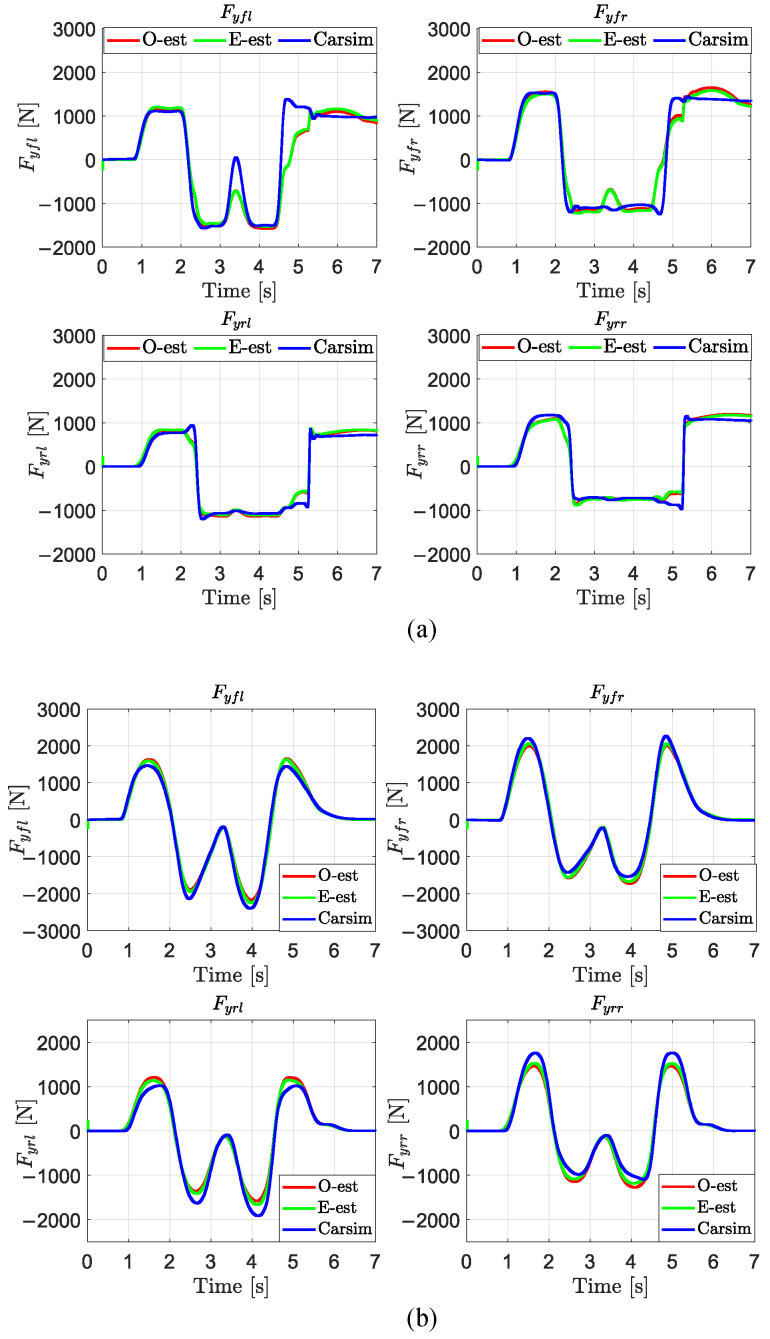

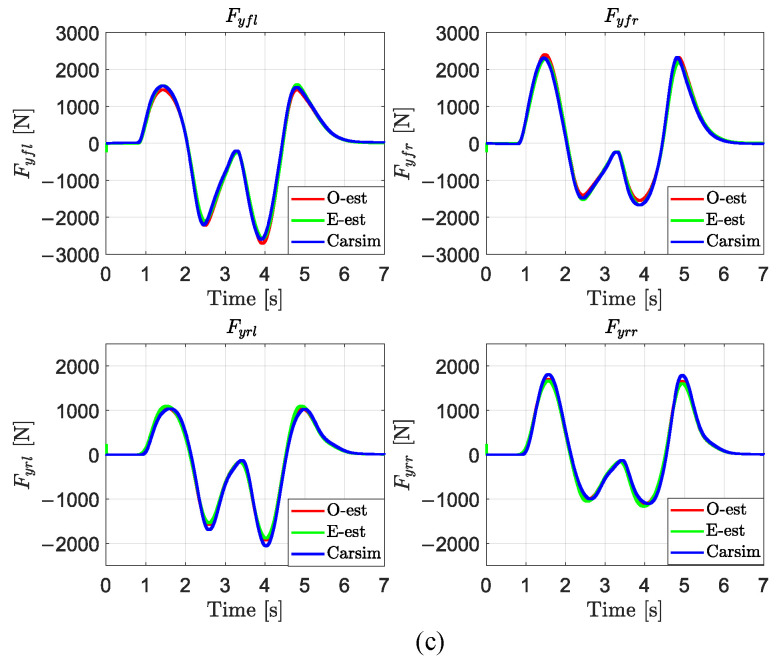
Estimation of $F_{yij}$ by different methods in the DLC test with $v_{x}$ = 80 km/h. (**a**) μ=0.3; (**b**) μ=0.5; (**c**) μ=0.85.

To investigate the impact of the accuracy of $F_{zij}$ in the upper-layer estimator on the estimation of $F_{yij}$ in the lower-layer estimator, this study designed two comparative schemes and conducted DLC tests on low-, medium-, and high-friction road surfaces. In the first scheme, $F_{zij}$ obtained from open-loop estimation was input into the lower-layer estimator to estimate $F_{yij}$, referred to as O-Eest. In the second scheme, $F_{zij}$ derived from the upper-layer estimator was input into the lower-layer estimator to estimate $F_{yij}$, using the method described previously and referred to as E-est. The results are shown in Table 6 and Figure 11 where $F_{yij}$ estimated by E-est is closer to the true values output by CarSim compared to O-Eest. Specifically, as shown in the data from Table 6, E-est achieves significantly higher accuracy than O-Eest, particularly on low-friction road surfaces. The corresponding MAE, ME, and RMSE values are improved by 46.67%, 40.21%, and 48.64%, respectively. It can be concluded that the accurate estimation of $F_{zij}$ has a significant impact on the estimation of $F_{yij}$, especially under low-friction conditions (μ=0.3).

**Figure 12:**
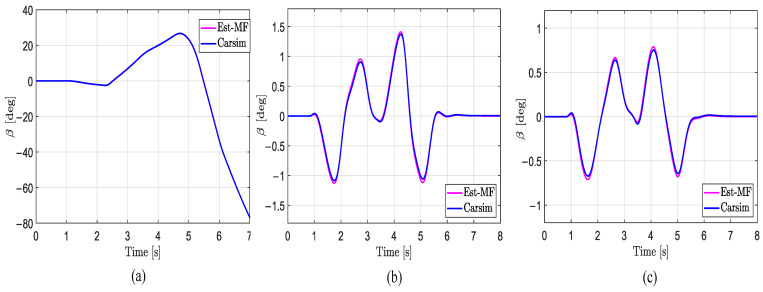
Estimation of β in the DLC test with $v_{x}$ = 80 km/h. (**a**) μ=0.3; (**b**) μ=0.5; (**c**) μ=0.85.

As shown in Figure 12 and Table 7, the proposed method can accurately estimate the β under low-, medium-, and high-road-adhesion conditions. Even on surfaces with μ=0.3, where the vehicle is in an unstable state, the proposed method still effectively estimates β. This demonstrates that the method used has reliable accuracy.

#### 3.4.3. Estimation Results of Cornering Stiffness

**Figure 13:**
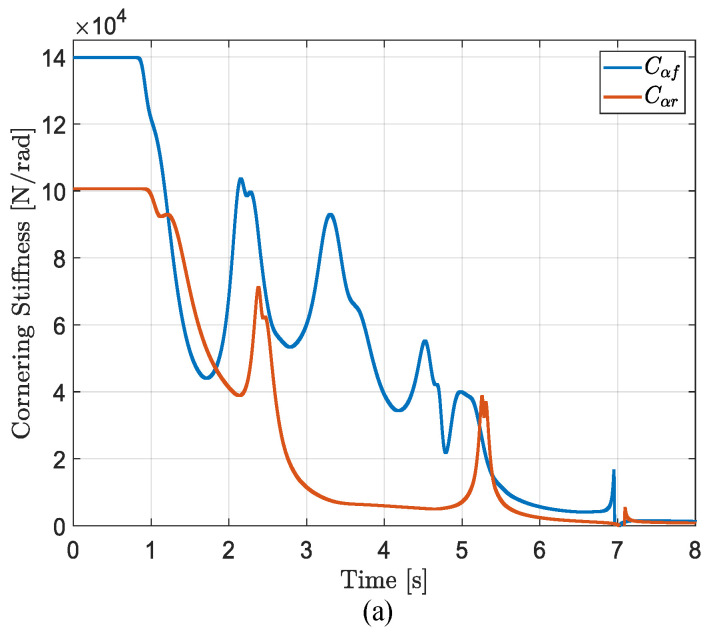

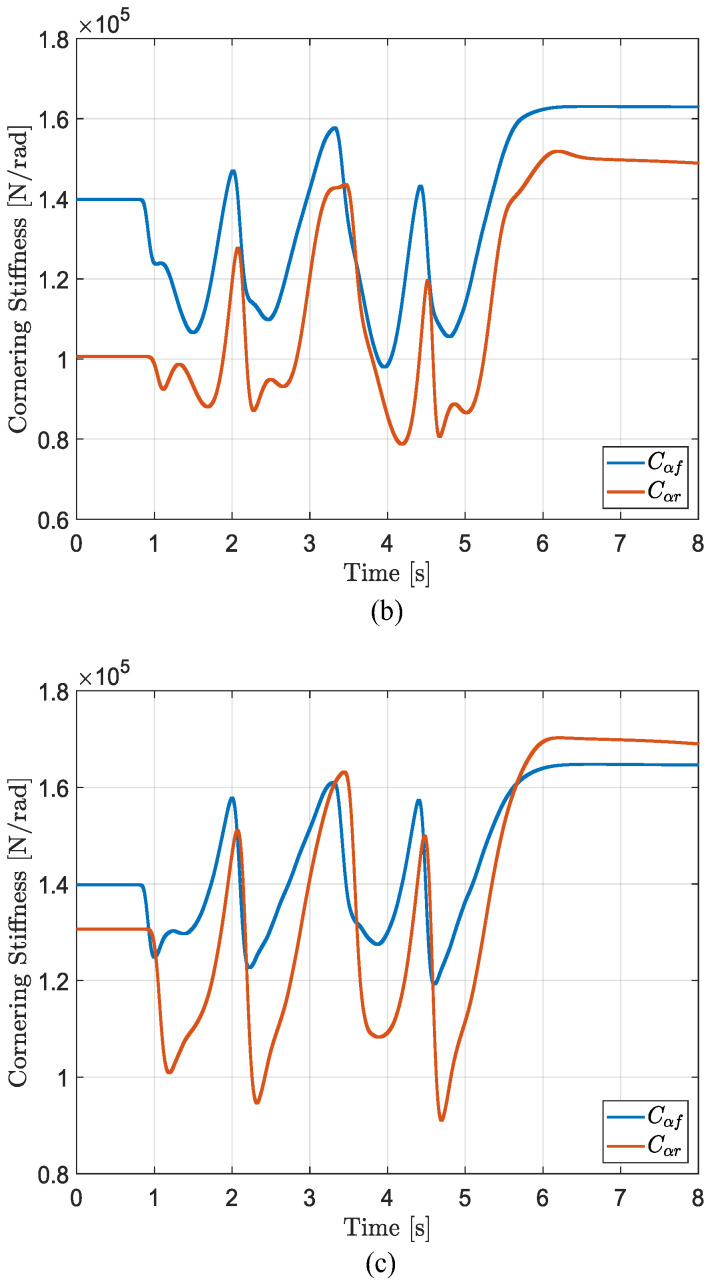
Estimation results of cornering stiffness in the DLC test with $v_{x}$ = 80 km/h. (**a**) μ=0.3; (**b**) μ=0.5; (**c**) μ=0.85.

The cornering stiffness estimation results are shown in Figure 13. From the three subplots, it can be observed that the vehicle’s cornering stiffness varies under different road conditions. During the period from 0 to 0.86 s, the vehicle is in straight-line driving, with no lateral force or slip angle, and the cornering stiffness values of the front and rear axles remain at their initial assigned values. At 0.86 s, the vehicle begins to steer, and the cornering stiffness changes due to factors such as mass transfer induced by steering. Combining Figure 9 and Figure 10, under low-adhesion conditions (μ=0.3), the insufficient lateral force provided by the road causes the vehicle to skid and lose stability, resulting in a decrease in the estimated cornering stiffness. On medium- and high-adhesion roads (μ=0.5 and μ=0.85), the vehicle remains in a stable state. The cornering stiffness of the front and rear axles changes with the actual lateral load transfer between the left and right sides of the vehicle and the maximum lateral force provided by the road. When the lateral load transfer on a given axle increases, the corresponding cornering stiffness decreases; conversely, when the load transfer decreases, the cornering stiffness increases. Similarly, the greater the maximum lateral force provided by the road, the larger the corresponding cornering stiffness; conversely, the smaller the maximum lateral force, the smaller the cornering stiffness.

## 4. Controller Design

**Figure 14:**
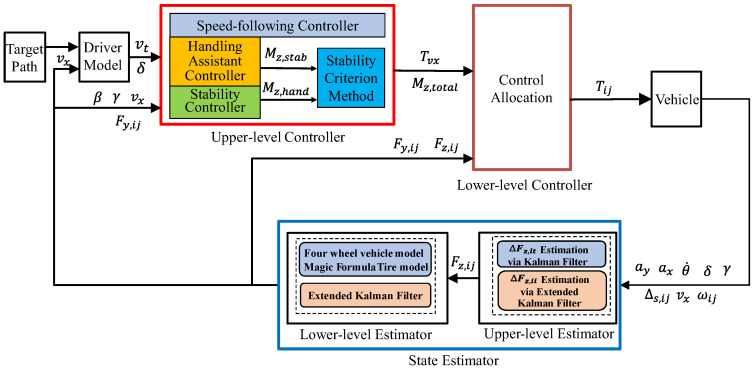
Block diagram of the overall control system.

Figure 14 shows a block diagram of the overall system. The control framework consists of an upper-level controller and a lower-level controller. The upper-layer controller generates the torques required to maintain vehicle stability or enhance handling, as well as the torques necessary for vehicle speed tracking. The lower-layer controller distributes the transmitted torques and forces to the four wheels. This study focuses on the design of a vehicle stability criterion method and the lower-layer allocation controller. Other components, such as the handling assistance controller, stability controller, and speed-following controller, are implemented based on the methods in [24].

### 4.1. Stability Criterion Method

This study refers to [25] and utilizes the understeer coefficient to determine vehicle stability. The understeer coefficient $K_{us}$ is expressed as shown in (49). Here, $C_{\alpha f}$ and $C_{\alpha r}$ are estimated using the method described in Section 3.3.

(49) $$K_{us}=\frac{m(l_{r}C_{\alpha r}-l_{f}C_{\alpha f})}{lC_{\alpha f}C_{\alpha r}}$$

When a vehicle is turning, its steering response characteristics can be classified into three types: Neutral Steer, Understeer, and Oversteer. The corresponding understeer coefficient $K_{us}$ exhibits the following properties for each type:

(50) $$K_{us}\left\{\begin{array}{l} >0,Understeer\\ =0,NeutralSteer\\ <0,Oversteer \end{array}\right.$$

**Figure 15:**
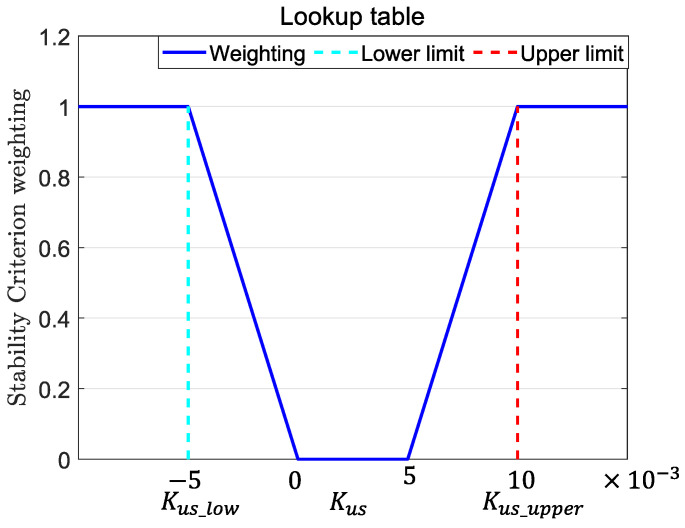
Vehicle stability control weight coefficient W.

For regular drivers, the ideal steering characteristic of a vehicle is neutral steer with a slight understeer tendency to ensure better handling and control. Referring to [25], this study uses $K_{us}$ to evaluate vehicle stability, with the absolute value of $K_{us\_upper}$ set to twice the absolute value of $K_{us\_low}$. This threshold can be adjusted based on individual driving preferences to meet different needs. Based on this, the upper-level controller determines the stability control weighting coefficient W, which is used to calculate the proportion of torque generated by the handling assist controller and the stability controller in the total torque output of the upper-level controller, as shown in (51). For different driving conditions, a lookup table is established, as shown in Figure 15, to set the critical values of $K_{us}$ for stability determination. When W=0, it indicates that the vehicle is stable, and additional torque is applied to improve handling. When W=1, the vehicle is unstable or experiencing severe understeer (front axle skidding), requiring torque to maintain stability. The required torque is applied to ensure the vehicle’s stability.

(51) $$M_{z,total}={\left(1-W\right)\cdot M}_{z,hand}{+W\cdot M}_{z,stab}$$

Here, $M_{z,total}$ represents the total torque transmitted from the upper-layer controller to the lower-layer controller. $M_{z,hand}$ is the additional torque generated by the handling assistance controller to improve vehicle handling, while $M_{z,stab}$ is the additional torque generated by the stability controller to maintain stable vehicle operation.

### 4.2. Control Allocation

In order to effectively distribute the additional yaw moment $M_{z,total}$ generated by the upper-level controller and the torque $T_{vx}$ required to maintain vehicle speed to the four wheels, the relationship is derived as shown in (52). Since (52) corresponds to an over-actuated system, optimal torque distribution to the four wheels is achieved using control allocation (CA). In this study, Quadratic Programming (QP) is employed, aiming to minimize the utilization of road adhesion by the four wheels. The optimal torque values allocated to each wheel are obtained by solving this optimization problem.

(52) $$\left\{\begin{array}{l} M_{z,total}=\frac{T_{fl}}{R}\left(-\frac{t_{f}}{2}cos\delta +l_{f}sin\delta \right)+\frac{T_{fr}}{R}\left(\frac{t_{f}}{2}cos\delta +l_{f}sin\delta \right)-\frac{T_{rl}t_{r}}{2R}+\frac{{T_{rr}t}_{r}}{2R}\\ \;T_{vx}=T_{fl}cos\delta +T_{fr}cos\delta +T_{rl}+T_{rr} \end{array}\right.$$

where $T_{ij}$ represents the torque applied to the corresponding wheel of the vehicle, R denotes the wheel’s radius.

(53) $$\sqrt{{\left(\frac{T_{x,ij}}{R}\right)}^{2}+{\left(F_{yij}\right)}^{2}}\leq \mu_{ij}F_{zij}$$

**Figure 16:**
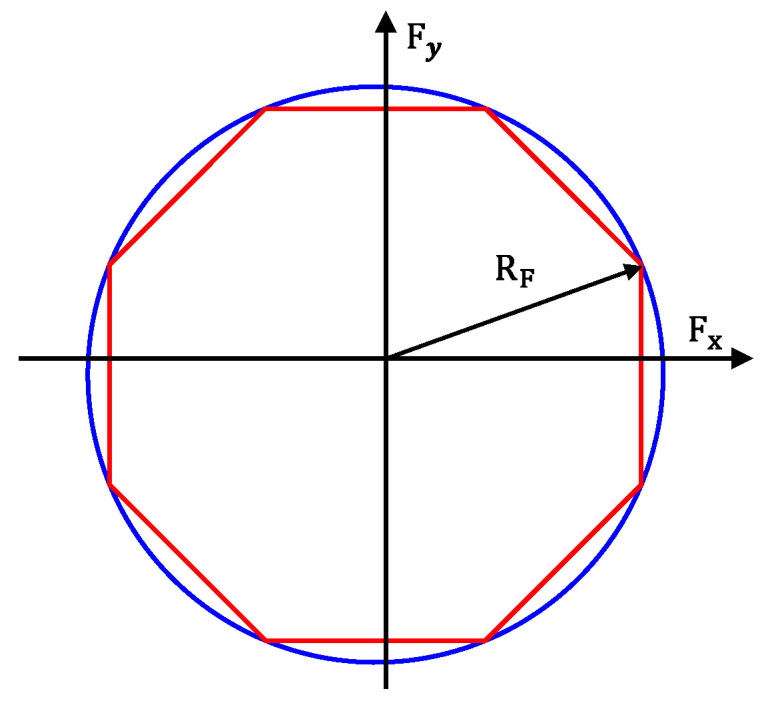
Linearization of the tire adhesion circle. Where, the red color represents the octagonal constraint, and the blue color represents the adhesion circle.

The forces acting on the wheels are constrained by the traction limit circle, as expressed in (53). Here, $\mu_{ij}$ represents the friction coefficient between the wheel and the road, while $F_{yij}$ and $F_{zij}$ denote the lateral and vertical forces on the wheel, respectively. Since (53) is a nonlinear inequality and challenging to solve directly, this study simplifies the traction limit circle into a linear octagon, as shown by the red octagon in Figure 16. In the figure, ${R_{F}=\mu \cdot F}_{zij}$, and the red octagon is inscribed within the blue traction limit circle. This simplification converts the nonlinear inequality in (53) into a linear inequality constraint problem, represented in (54).

(54) $$\begin{array}{c} {-\sqrt{2}\times {cos(22.5^{{}^{\circ}})\times \mu \cdot F}_{zij}-F_{yij}\leq F}_{xij}\leq \sqrt{2}\times {cos(22.5^{{}^{\circ}})\times \mu \cdot F}_{zij}-F_{yij}\\ {-{cos(22.5^{{}^{\circ}})\times \mu \cdot F}_{zij}\leq F}_{xij}\leq {cos(22.5^{{}^{\circ}})\times \mu \cdot F}_{zij} \end{array}$$

Assuming the adhesion coefficient for all four wheels is μ, the objective function can be expressed as shown in (55). Due to the coupling effects among the tire’s longitudinal, lateral, and vertical forces, to reduce computational complexity, this study focuses only on the optimization of the tire’s longitudinal forces. Consequently, the objective function is simplified to (56).

(55) $$J=min\sum \frac{1}{{\left({\mu \cdot F}_{zij}\right)}^{2}}\left[{\left(F_{xij}\right)}^{2}+{\left(F_{yij}\right)}^{2}\right]$$

(56) $$J=min\sum \frac{1}{{\left({\mu \cdot F}_{zij}\right)}^{2}}\left[{\left(F_{xij}\right)}^{2}\right]$$

The quadratic objective function of the optimization problem can be formulated based on (52), (54), and (56), as expressed in (57):

(57) $$\min\frac{1}{2}T_{x}^{T}HT_{x},subject\;to\left\{\begin{array}{c} A_{eq}\cdot T_{x}=b_{eq}\\ A\cdot T_{x}\leq b \end{array}\right.$$

where $A_{eq}$ is the control matrix that describes the relationship between the command vector $b_{eq}$ and the input vector $T_{x}$ and Matrix A describes the linear inequality constraint relationship between the input vector $T_{x}$ and the command vector b.

$$A_{eq}=\left[\begin{array}{cccc} \frac{1}{R}(-\frac{t_{f}}{2}cos\delta +l_{f}sin\delta ) & \frac{1}{R}(\frac{t_{f}}{2}cos\delta +l_{f}sin\delta ) & -\frac{t_{r}}{2R} & \frac{t_{r}}{2R}\\ cos\delta & cos\delta & 1 & 1 \end{array}\right],b_{eq}=\left[\begin{array}{c} M_{z,total}\\ T_{vx} \end{array}\right]$$

$$T_{x}={\left[\begin{array}{cccc} T_{fl} & T_{fr} & T_{rl} & T_{rr} \end{array}\right]}^{T},A=\left[\begin{array}{c} A1\\ A2 \end{array}\right],b=\left[\begin{array}{c} b1\\ b2 \end{array}\right]$$

$$A1=\frac{1}{R}\times \left[\begin{array}{c} \begin{array}{cccc} 1 & 0 & 0 & 0\\ -1 & 0 & 0 & 0 \end{array}\\ \begin{array}{cccc} 0 & 1 & 0 & 0\\ 0 & -1 & 0 & 0 \end{array}\\ \begin{array}{cccc} 1 & 0 & 0 & 0\\ -1 & 0 & 0 & 0 \end{array}\\ \begin{array}{cccc} 1 & 0 & 0 & 0\\ -1 & 0 & 0 & 0 \end{array} \end{array}\right],A2=\frac{1}{R}\times \left[\begin{array}{c} \begin{array}{cccc} 1 & 0 & 0 & 0\\ -1 & 0 & 0 & 0 \end{array}\\ \begin{array}{cccc} 0 & 1 & 0 & 0\\ 0 & -1 & 0 & 0 \end{array}\\ \begin{array}{cccc} 1 & 0 & 0 & 0\\ -1 & 0 & 0 & 0 \end{array}\\ \begin{array}{cccc} 1 & 0 & 0 & 0\\ -1 & 0 & 0 & 0 \end{array} \end{array}\right],b1=\frac{1}{R}\times \left[\begin{array}{c} \cos\left(22.5\right){\mu \cdot F}_{zfl}\\ \cos\left(22.5\right){\mu \cdot F}_{zfl}\\ \cos\left(22.5\right){\mu \cdot F}_{zfr}\\ \cos\left(22.5\right){\mu \cdot F}_{zfr}\\ \cos\left(22.5\right){\mu \cdot F}_{zrl}\\ \cos\left(22.5\right){\mu \cdot F}_{zrl}\\ \cos\left(22.5\right){\mu \cdot F}_{zrr}\\ \cos\left(22.5\right){\mu \cdot F}_{zrr} \end{array}\right]$$

$$b2=\frac{1}{R}\times \left[\begin{array}{c} \sqrt{2}\cos\left(22.5\right){\mu \cdot F}_{zfl}-F_{yfl}\\ \sqrt{2}\cos\left(22.5\right){\mu \cdot F}_{zfl}-F_{yfl}\\ \sqrt{2}\cos\left(22.5\right){\mu \cdot F}_{zfr}-F_{yfr}\\ \sqrt{2}\cos\left(22.5\right){\mu \cdot F}_{zfr}-F_{yfr}\\ \sqrt{2}\cos\left(22.5\right){\mu \cdot F}_{zrl}-F_{yrl}\\ \sqrt{2}\cos\left(22.5\right){\mu \cdot F}_{zrl}-F_{yrl}\\ \sqrt{2}\cos\left(22.5\right){\mu \cdot F}_{zrr}-F_{yrr}\\ \sqrt{2}\cos\left(22.5\right){\mu \cdot F}_{zrr}-F_{yrr} \end{array}\right],H=\frac{1}{{\left(\mu R\right)}^{2}}\left[\begin{array}{cccc} \frac{1}{{\left(F_{zfl}\right)}^{2}} & 0 & 0 & 0\\ 0 & \frac{1}{{\left(F_{zlr}\right)}^{2}} & 0 & 0\\ 0 & 0 & \frac{1}{{\left(F_{zrl}\right)}^{2}} & 0\\ 0 & 0 & 0 & \frac{1}{{\left(F_{zrr}\right)}^{2}} \end{array}\right]$$

## 5. Results

The designed controller was built in MATLAB and Simulink (version 2023b) and co-simulated with CarSim to conduct DLC tests.

**Figure 17:**
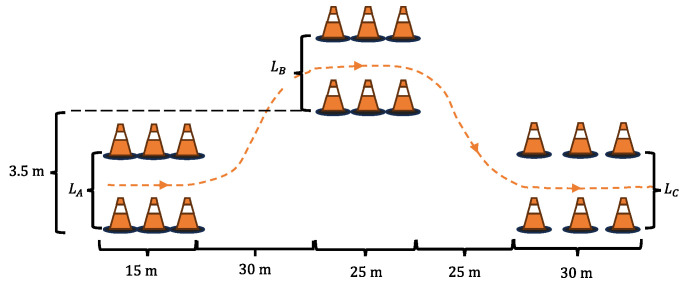
Track layout for ISO 3888-1. $L_{A}=1.1\times W_{v}+0.25;\;L_{B}=1.2\times W_{v}+0.25;\;L_{C}=1.3\times W_{v}+0.25;$ $W_{v}$ is the vehicle width.

The DLC test is a closed-loop testing method configured according to the ISO 3888-1 international standard [26], as shown in Figure 17. In the test, the driver model provided by CarSim was utilized with a preview time of 0.58 s, and simulations were carried out at a vehicle speed of $v_{x}=100\;km/h$ under low (μ=0.3) and medium (μ=0.5) road adhesion conditions. The lateral forces $F_{yij}$ and vertical loads $F_{zij}$ estimated using the open-loop method were compared with the results obtained from the estimator designed in this study. The open-loop estimation is denoted as O-Est, the estimator designed in this study is denoted as Est, and the direct outputs from CarSim are referred to as Carsim. A comparative analysis of the results was performed.

**Figure 18:**
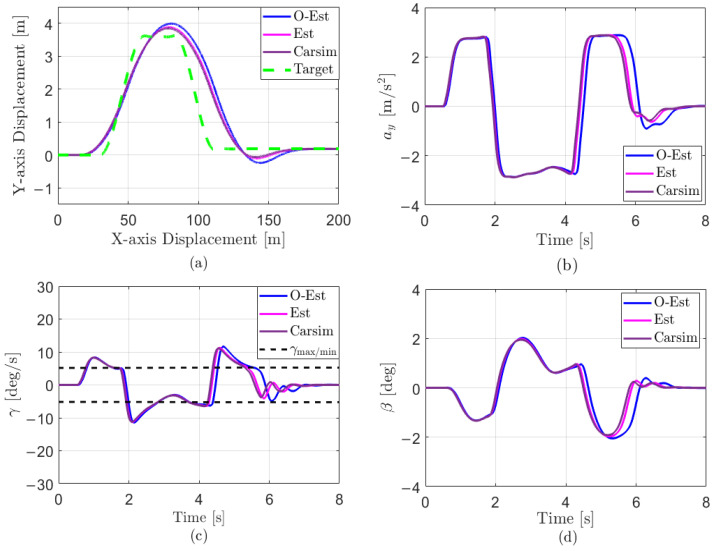
Results of the DLC test with $v_{x}=100\;km/h$ and μ=0.3. (**a**) Vehicle trajectories; (**b**) lateral acceleration response; (**c**) yaw rate response; (**d**) sideslip angle response.

**Figure 19:**
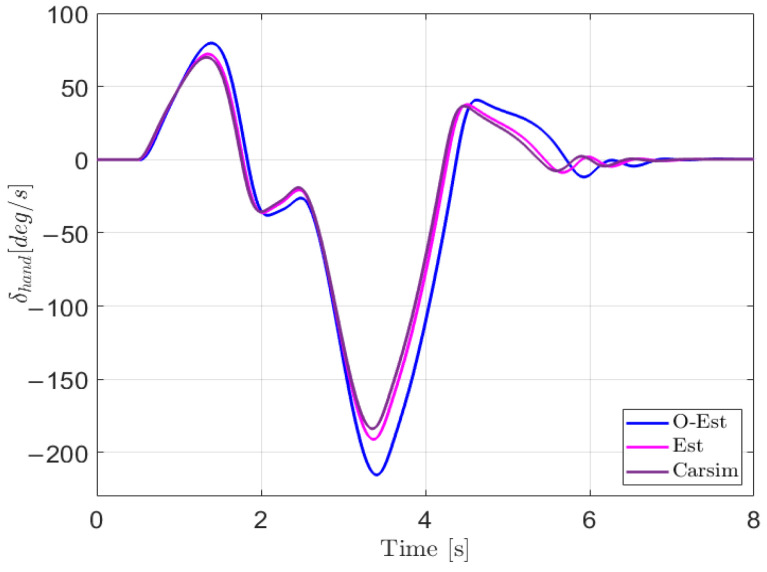
Driver model input in the DLC test with $v_{x}=100\;km/h$ and μ=0.3.

**Figure 20:**
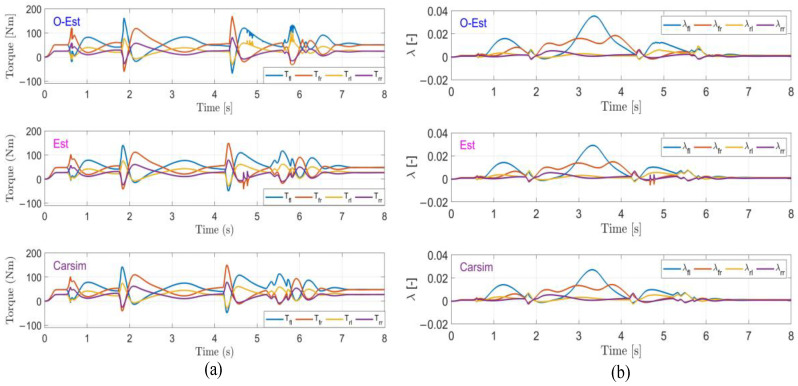
Results of the DLC test with $v_{x}=100\;km/h$ and μ=0.3. (**a**) The torque curve; (**b**) the slip ratio curve.

The results of the test under low-adhesion conditions (μ=0.3) are shown in Figure 18. From the driving trajectory in Figure 18a and the lateral acceleration in Figure 18b, it can be observed that under the controller’s operation, all three test scenarios successfully completed the lane change stably, with lateral accelerations remaining below the maximum values provided by the road surface, indicating no vehicle instability. However, due to the high-speed lane change on a low-adhesion surface and the limited lateral force provided by the road, all three test scenarios showed some deviation from the target path. The O-Est group exhibited larger deviations from the target path compared to the Est and Carsim groups and took longer to return to stable driving after completing the lane change. From Figure 19 and Figure 20, it is evident that the O-Est group introduced significant errors in estimating $F_{yij}$ and $F_{zij}$, especially during intense vehicle motion. These errors resulted in inaccuracies when the controller calculated the torque distribution to the four wheels, causing the total torque generated by the lower-layer wheels to deviate from the required torque provided by the upper layer. This led to greater deviations in the vehicle’s trajectory. Table 8 provides an intuitive explanation for why the O-Est group performed worse in following the target path compared to the other two groups. The larger deviation from the target path in the O-Est group caused higher maximum values of β and γ compared to the other groups. This required larger additional torques to maintain vehicle stability and handling, resulting in higher torque distribution to the four wheels. Consequently, the slip ratio of the wheels increased, reducing the vehicle’s lateral stability reserve. To compensate and guide the vehicle back to the target path, the driver model applied a larger steering angle input $\delta_{hand}$, with a maximum value reaching 215.21 degrees. The total time for three test groups was obtained using the Profile tool. Among them, the Carsim group had the shortest runtime of 7.446 s, while the Est group was slightly longer at 7.726 s. All three test execution times were less than 8 s. During the execution, a total of 96,013 calls were made, with each call taking less than $8.332\times 10^{-5}$ s, which is significantly shorter than the computation cycle of mainstream controllers ($5\times 10^{-3}$ s). Therefore, the designed controller operates stably and reliably, meeting the requirements for real-time control.

**Figure 21:**
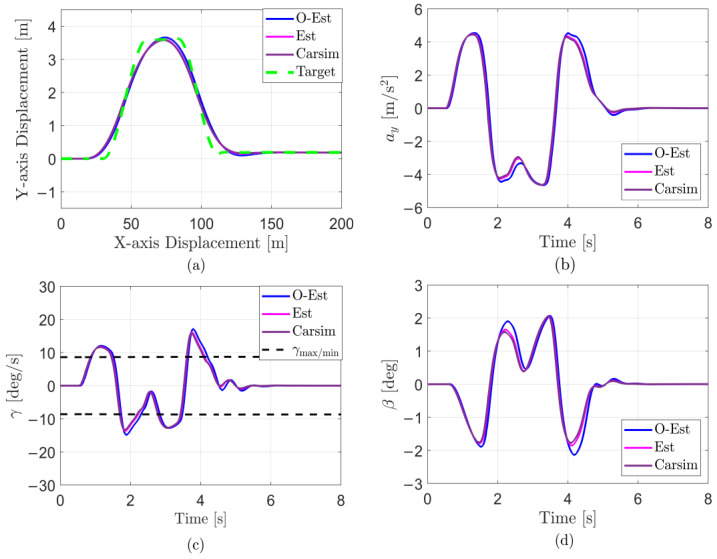
Results of the DLC test with $v_{x}=100\;km/h$ and μ=0.5. (**a**) Vehicle trajectories; (**b**) lateral acceleration response; (**c**) yaw rate response; (**d**) sideslip angle response.

**Figure 22:**
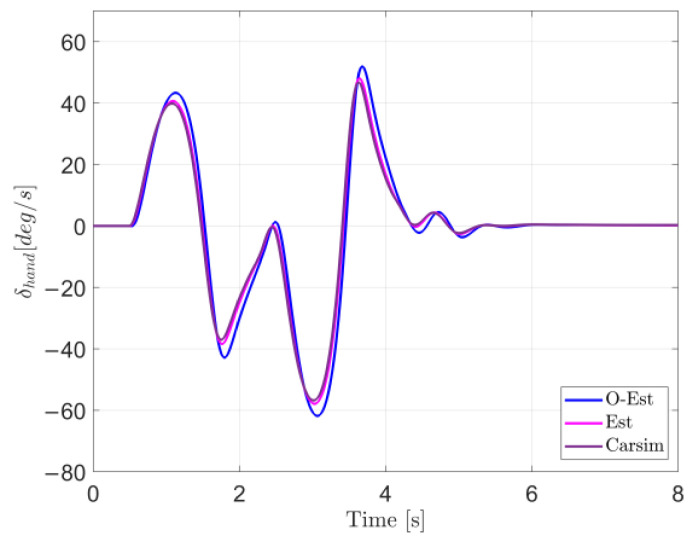
Driver model input in the DLC test with $v_{x}=100\;km/h$ and μ=0.5.

**Figure 23:**
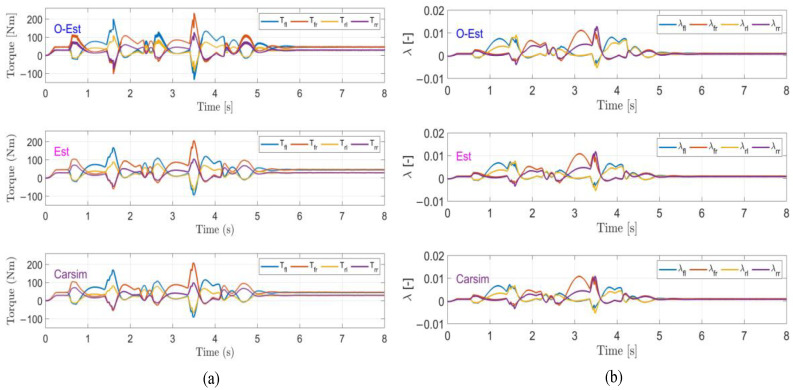
Results of the DLC test with $v_{x}=100\;km/h$ and μ=0.5. (**a**) The torque curve; (**b**) the slip ratio curve.

Under medium-adhesion conditions (μ=0.5), the test results are shown in Figure 21. From the trajectory diagram in Figure 21a and the lateral acceleration in Figure 21b, it can be observed that, under the controller’s operation, all three test scenarios stably and closely followed the target path during the lane change, with lateral accelerations remaining below the maximum values provided by the road surface, indicating no vehicle skidding or instability. At 2 s and 4 s, Figure 21b–d show that the O-Est group exhibits larger lateral acceleration, yaw rate, and sideslip angle compared to the Est and Carsim groups. Combining Figure 22 and Figure 23, it is evident that when the vehicle’s lateral acceleration exceeds 0.3 g, the vehicle enters a state of intense motion. The O-Est group introduces errors in estimating $F_{yij}$ and $F_{zij}$, leading to inaccuracies when the controller calculates the torque distribution to the four wheels. This results in a mismatch between the total torque generated by the lower-layer wheels and the required torque from the upper layer, causing larger deviations in the vehicle’s trajectory. Table 9 provides an intuitive explanation for the O-Est group’s deviation from the target path compared to the other two groups. During intense vehicle motion, the O-Est group shows larger maximum values of β and γ than the other two groups. This necessitates higher additional torques to maintain vehicle stability and handling, resulting in higher torque distribution to the four wheels. Consequently, the slip ratio of the wheels increases, reducing the vehicle’s lateral stability reserve. To compensate and guide the vehicle back to the target path, the driver model applied a larger steering angle input $\delta_{hand}$, with a maximum value reaching 61.83 degrees. The total time for three test groups on medium-adhesion road surfaces was obtained using the Profile tool. Among them, the Carsim group had the shortest runtime of 7.443 s, while the Est group was slightly longer at 7.726 s. All three test execution times were less than 8 s. During the execution, a total of 96,013 calls were made, with each call taking less than $8.332\times 10^{-5}$ s, which is significantly shorter than the computation cycle of mainstream controllers ($5\times 10^{-3}$ s). Therefore, the designed controller operates stably and reliably, meeting the real-time control requirements on both medium- and low-road-adhesion surfaces.

## 6. Conclusions

This study obtained the following key findings:

A hierarchical estimator was designed. Based on data collected from commonly used onboard sensors, a vehicle state estimation equation was established using a 3-DOF four-wheel vehicle model combined with the Magic Formula Tire model to estimate vehicle states in real time. Closed-loop DLC simulations were conducted under low-, medium-, and high-road-adhesion conditions. The results demonstrate that the proposed hierarchical estimation method is more time-efficient and achieves higher accuracy.

A hierarchical control system for lateral stability was designed. The upper layer determines stability requirements based on driver inputs and vehicle states, dynamically switching between handling assistance mode and stability control mode. It generates the required yaw moment and speed control torque, which are transmitted to the lower layer. The lower layer linearizes the nonlinear constraints and employs control allocation methods, utilizing Quadratic Programming (QP) to achieve optimal torque distribution to the four wheels.

The hierarchical estimator proposed in this paper was executed on a laboratory computer, and its runtime performance on an in-vehicle computer remains to be further verified through real vehicle testing. Additionally, the road adhesion coefficient is another critical research area in vehicle stability controller design. The testing scenarios in this study were conducted under known road adhesion conditions, lacking an estimation of the road adhesion coefficient. Future research on the controller should focus on accurately estimating the road adhesion coefficient in real time.

## Figures and Tables

**Table 1:** Fitted parameters of the MF-T.

F	$a_{1}$	$a_{2}$	$a_{3}$	$a_{4}$	$a_{5}$	$a_{6}$	$a_{7}$	$a_{8}$	C
$F_{x}$	−9.105	1017	969.3	$3.206\times 10^{4}$	0.04033	$1.094\times 10^{-17}$	$-1.198\times 10^{-16}$	0.55	1.4
$F_{y}$	−9.089	1017	9730	0.3505	0.09514	$1.317\times 10^{-19}$	$-1.1941\times 10^{-18}$	−0.2752	1.413

**Table 2:** Main parameter values of C-class hatchback.

Parameter	Value
Total vehicle weight m (kg)	1592.00
Sprung mass $m_{s}$ (kg)	1230.00
Vehicle track width E (mm)	1675.00
Wheelbase l (mm)	2600.00
Distance from the CG to the front axle $l_{f}$ (mm)	1065.00
Distance from the CG to the rear axle $l_{r}$ (mm)	1535.00
Height of the CG above the ground h (mm)	540.00
Yaw moment of inertia $I_{z}\;(kg\cdot m^{2})$	1520.00

**Table 3:** Estimation error of $∆F_{zl}$ in DLC test.

μ	MAE (N)	ME (N)	RMSE (N)
0.3	124.535	534.431	145.243
0.5	106.282	297.750	150.768
0.85	92.349	285.897	126.757

**Table 4:** Estimation error of $F_{zij}$ under DLC test.

μ	Case	MAE (N)	ME (N)	RMSE (N)	Total Time (s)
0.3	O-Est	163.28	372.66	184.70	0.042
	Est	66.40	206.51	87.69	0.095
0.5	O-Est	72.27	324.86	102.88	0.044
	Est	49.15	166.73	61.68	0.096
0.85	O-Est	56.69	280.15	101.41	0.042
	Est	36.98	112.91	50.23	0.096

**Table 5:** Estimation error of $F_{yij}$ in DLC test.

μ	Case	MAE (N)	ME (N)	RMSE (N)	Total Time (s)
0.3	O-Est	143.03	1282.69	274.26	0.069
	Est	70.56	512.96	109.94	0.188
0.5	O-Est	163.31	1019.84	262.42	0.073
	Est	62.17	349.07	95.92	0.187
0.85	O-Est	172.31	784.44	251.82	0.068
	Est	57.06	199.02	78.60	0.189

**Table 6:** Estimation error of $F_{yij}$ by different methods under DLC test.

μ	Case	MAE (N)	ME (N)	RMSE (N)	Total Time (s)
0.3	O-est	132.317	857.882	214.054	0.135
	E-est	70.564	512.962	109.938	0.188
0.5	O-est	88.625	387.789	130.725	0.137
	E-est	62.170	349.067	95.924	0.187
0.85	O-est	60.040	223.918	82.959	0.135
	E-est	57.061	199.022	78.601	0.189

**Table 7:** Estimation error of β in DLC test.

μ	MAE (deg)	ME (deg)	RMSE (deg)	Total Time (s)
0.3	$6.99\times 10^{-2}$	0.28	$10.09\times 10^{-2}$	0.188
0.5	$1.68\times 10^{-2}$	0.06	$2.34\times 10^{-2}$	0.187
0.85	$1.31\times 10^{-2}$	0.05	$1.79\times 10^{-2}$	0.189

**Table 8:** Comparison of control metrics and input parameters in DLC test ($v_{x}=100\;km/h,\mu =0.3$).

Case	$\beta_{max}(deg)$	$r_{max}(deg/s)$	$T_{max}(Nm)$	$\lambda_{max}(-)$	$\delta_{handmax}(deg)$	Total Time (s)
O-Est	2.07	11.64	170.93	3.55%	215.21	7.554
Est	1.98	11.28	149.68	2.92%	190.91	7.726
Carsim	1.96	11.22	145.25	2.72%	183.61	7.446

**Table 9:** Comparison of control metrics and input parameters in DLC test ($v_{x}=100\;km/h,\mu =0.5$).

Case	$\beta_{max}(deg)$	$r_{max}(deg/s)$	$T_{max}(Nm)$	$\lambda_{max}(-)$	$\delta_{handmax}(deg)$	Total Time (s)
O-Est	2.13	17.09	233.34	1.29%	61.83	7.554
Est	2.06	16.14	206.63	1.18%	57.9	7.726
Carsim	2.05	15.81	203.56	1.09%	56.77	7.443

## Data Availability

Data are contained within the article.
